# Microglial GRB2 is essential for brain ventriculogenesis and cerebrospinal fluid homeostasis

**DOI:** 10.1172/JCI185791

**Published:** 2026-09-10

**Authors:** Phan Q. Duy, Benjamin C. Reeves, Huanxing Sun, Xueyan Peng, Pazhanichamy Kalailingam, Garrett Allington, Evan Dennis, Le Thi Hao, Lei Wang, David Rufino-Ramos, Shujuan Zhao, Qiang Li, Neel H. Mehta, William C. Davalan, Mason Blacker, Anthony J. Piscopo, Shozeb Haider, Baojian Fan, Kedous Y. Mekbib, Shuai Shao, Carol Nelson-Williams, Tukiet T. Lam, Benjamin P. Kleinstiver, Patricia L. Musolino, Seth L. Alper, Sheng Chih Jin, Erica L. Herzog, Kristopher T. Kahle

**Affiliations:** 1 Department of Neurosurgery,; 2 Department of Neurology, and; 3 Department of Pediatrics, Massachusetts General Hospital, Harvard Medical School, Boston, Massachusetts, USA.; 4 Department of Neurosurgery, Yale University School of Medicine, New Haven, Connecticut, USA.; 5 Department of Neurosurgery, Barrow Neurological Institute, Phoenix, Arizona, USA.; 6 Section of Pulmonary, Critical Care, and Sleep Medicine, Department of Internal Medicine, Yale School of Medicine, New Haven, Connecticut, USA.; 7 Center for Genomic Medicine, Massachusetts General Hospital, Boston, Massachusetts, USA.; 8 Department of Neurology, Columbia University Vagelos College of Physicians and Surgeons, New York, New York, USA.; 9 Department of Pediatrics, Yale University School of Medicine, New Haven, Connecticut, USA.; 10 Department of Neuroscience, University of Virginia School of Medicine, Charlottesville, Virginia, USA.; 11 Department of Pathology, Massachusetts General Hospital, Boston, Massachusetts, USA.; 12 Department of Pathology, Harvard Medical School, Boston, Massachusetts, USA.; 13 Department of Genetics, School of Medicine, and; 14 Department of Pediatrics, School of Medicine, Washington University, St. Louis, Missouri, USA.; 15 School of Pharmacy, University College London, London, United Kingdom.; 16 Department of Genetics, Yale University School of Medicine, New Haven, Connecticut, USA.; 17 Department of Molecular Biophysics and Biochemistry, Yale University, New Haven, Connecticut, USA.; 18 Keck MS and Proteomics Resource, Yale School of Medicine, New Haven, Connecticut, USA.; 19 Division of Nephrology and Center for Vascular Biology Research, Beth Israel Deaconess Medical Center, and Department of Medicine, Harvard Medical School, Boston, Massachusetts, USA.; 20 Broad Institute of Harvard and MIT, Cambridge, Massachusetts, USA.; 21 Harvard Center for Hydrocephalus and Neurodevelopmental Disorders, Massachusetts General Hospital, Boston, Massachusetts, USA.

**Keywords:** Genetics, Neuroscience, Molecular genetics, Neurodevelopment

## Abstract

Microglia play essential yet poorly understood roles in brain development, including axon guidance, regulation of neurogenesis, and pruning of neuronal projections. Congenital hydrocephalus (CH), characterized by enlarged ventricles filled with cerebrospinal fluid (CSF), is a leading cause of pediatric brain surgery, but its molecular mechanisms remain unclear. We have identified what we believe to be novel, recurrent, damaging missense variants in the SH3-binding domain of the adaptor protein growth factor receptor-bound protein 2 (*GRB2*) in unrelated patients with CH. GRB2 is significantly coexpressed with 1 of its known upstream RTK partners, CSF1R, in the developing human brain, particularly in a microglial subtype associated with regulation of neural stem cells. IP validated GRB2-CSF1R binding in mouse microglial cells and a human monocyte cell line. *Cx3cr1-Grb2^fl/fl^* mice engineered with conditional deletion of *Grb2* in microglia exhibit congenital absence of microglia and early postnatal severe communicating (nonobstructive) hydrocephalus, mimicking the condition of patients with the *GRB2* mutation. The severe ventriculomegaly of *Cx3cr1-Grb2^fl/fl^* mice is associated with both depletion of cerebral cortical neurons and impairment of glia-lymphatic-mediated CSF flow. Together, these findings implicate a role of GRB2 in microglia that could be essential for brain development and CSF homeostasis.

## Abstract 2

To shed light on the molecular and genetic mechanisms of hydrocephalus, we studied multiple novel, damaging variants in the adaptor protein Growth Factor Receptor-Bound Protein 2 (GRB2).

## Introduction

The mammalian brain is populated with specialized mononuclear phagocytes known as microglia (1). These cells originate from primitive macrophages that emerge in the embryonic yolk sac to then enter the CNS before formation of the blood-brain barrier (2). As brain-resident macrophages, microglia perform numerous well-known immune functions, including CNS surveillance, antigen presentation, cytokine and chemokine production, and clearance of toxins and apoptotic cell debris. Recent research attributes additional functions to microglia related to CNS development, homeostasis, and repair, including guidance of growing axons, elimination of neuronal projections (“synaptic pruning”), regulation of neurogenesis and synaptogenesis, and support of neuronal survival during early postnatal development. However, the molecular mechanisms underlying many of these diverse functions and the connection between microglial dysfunction and neurodevelopmental disease (NDD) remain poorly understood.

Congenital hydrocephalus (CH), characterized by the primary perinatal enlargement of the cerebroventricular system, is the most common indication for neurosurgical intervention in children and is often associated with severe neurodevelopmental morbidity (3–5). Current treatment relies on neurosurgical cerebrospinal fluid (CSF) diversion through either shunt placement or endoscopic third ventriculostomy. Both treatments are accompanied by high rates of nonresponse, failure, and complications (3). The lack of alternative nonsurgical treatments reflects, to a large degree, our limited understanding of CH pathophysiology. Although CH has long been considered a primary disorder of impaired CSF dynamics (5), recent whole-exome sequencing (WES) studies have identified a significant subset of patients with CH with damaging mutations in various regulators of pre- or perinatal neurogenesis, suggesting that such cases may result from dysfunctional neurodevelopment rather than a primary CSF disturbance per se (4, 6–8). However, most cases of sporadic CH have no known genetic cause. More extensive understanding of CH genetics and pathophysiology could lead to improved prognosis and to personalized treatment strategies.

Growth factor receptor-bound protein 2 (GRB2) is an adaptor protein that binds to phosphorylated tyrosine residues on upstream RTKs to facilitate downstream RAS activation (9). One of the most important GRB2-binding RTKs is the colony-stimulating factor 1 receptor (CSF1R), which is highly expressed on microglia, macrophages, and osteoclasts (9, 10). CSF1-regulated CSF1R signaling in the CNS controls proliferation, differentiation, and survival of microglia, as well as of other CNS-resident macrophages of the meninges, choroid plexus, and perivascular spaces (11). *Csf1r* KO in mice results in absence of brain microglia, severe perinatal hydrocephalus, and other structural brain abnormalities (10). Human *CSF1R* mutations similarly result in NDDs, encephalopathies (e.g., hereditary diffuse leukoencephalopathy, degenerative encephalopathy), and other structural brain defects, including cerebral ventriculomegaly (CV) and shunt-dependent CH (12–14). In contrast, germline variants in *GRB2* have not been linked to any human disease. However, understanding the roles of GRB2 in microglia and brain development has been hindered by embryonic lethality of *Grb2* KO mice, reflecting defective endodermal cell differentiation and epiblast formation (15). Understanding the role of GRB2 in ventriculogenesis may yield additional insights into the molecular pathways that regulate brain development, CSF homeostasis, and pathogenic events leading to hydrocephalus.

In this study, we report our identification of previously unreported, recurrent, damaging missense variants in the GRB2 SH3 domain in patients with CH. We also show that conditional *Grb2* deletion in mouse microglia results in severe perinatal communicating hydrocephalus, the absence of brain microglia, reduced numbers of cortical neurons, and impaired CSF flux through the glia-lymphatic (“glymphatic”) system. These data implicate an essential role for microglial GRB2 in brain development, CSF homeostasis, and human disease.

## Results

### Variants in the C-terminal SH3 domain of GRB2 in human CH.

Our analysis of WES data from our cohort of 482 probands with sporadic CH treated by neurosurgical CSF diversion (i.e., either ventriculo-peritoneal CSF shunting or endoscopic third ventriculostomy; see Methods) identified recurrent, previously unreported, damaging germline missense variants in the C-terminal SH3 domain of GRB2 (encoded by *GRB2*) at highly conserved, adjacent aa positions (KCHYD65-1 [p.R178C] and KCHYD52-1 [p.R179Q]) (Figure 1, A and B, Supplemental Figure 1A, and Table 1; supplemental material available online with this article; https://doi.org/10.1172/JCI185791DS1). Patients KCHYD65-1 and KCHYD52-1 had unaffected transmitting parents and had shunt-dependent communicating hydrocephalus without other obvious structural brain malformations (Figure 1A). Rare, heterozygous, damaging variants in *GRB2*, transmitted or unphased, were identified in 2 additional patients from the Gabriella Miller KidsFirst (GMKF) database who had CV and neurodevelopmental delay (Table 1), including a second identical p.R178C variant in an unrelated proband and the variant p.R215Q (Figure 1B and Table 1).

GRB2 is an adaptor protein containing 1 SH2 domain and 2 SH3 domains (Figure 1B). The GRB2 SH2 domain binds tyrosine-phosphorylated sequences in the upstream RTKs, such as CSF1R (9), and its 2 SH3 domains direct complex formation with proline-rich regions of downstream signaling proteins such as the tyrosine phosphatase SHP2 (Figure 1C) (16–19). In silico biophysical modeling localized p.R178C and p.R179Q to the C-terminal SH3 domain of GRB2, where the 2 mutant residues are predicted to disrupt interactions between the guanidinium side chains of WT GRB2 R178/R179 and the carbonyl oxygens of SHP2 residues T564, P565, and P568 (Figure 1, D and E).

p.R215Q, also localized to the C-terminal SH3 binding domain of GRB2, is predicted to impair downstream GRB2 signaling by disrupting 2 hydrogen-bond interactions with the backbone carbonyl oxygen atoms of Q157 and P158, reducing loop-region flexibility and indirectly destabilizing adjacent residue Y160, required for constitutive GRB2 phosphorylation (Supplemental Figure 1B). These data identify multiple previously unreported or rare, damaging missense variants in the C-terminal SH3 domain of GRB2 in patients with CH.

### CH-associated variants in a CSF1R/GRB2/SHP2 signaling network.

Based on these findings with GRB2, we investigated whether other patients with CH in these cohorts carried damaging variants in GRB2-binding partners CSF1R or SHP2 (Figure 1F and Supplemental Figure 1, C and D). Mouse models expressing Csf1r mutants (10, 20–22) or Shp2 mutants (20) are both associated with severe CH. Patients with variants in CSF1R (21, 22) or SHP2 (23–25) have also been described with CV or CH. *CSF1R* contained a heterozygous transmitted loss-of-function variant (p.R216X) in KCHYD28-1 predicted to truncate the CSF1R C-terminal phospho-tyrosine domain, which binds and activates GRB2 (Supplemental Figure 1C and Table 1) (26–28). *CSF1R* also contained 3 other previously unreported or rare, damaging, transmitted or unphased heterozygous missense variants, each located in the CSF1R C-terminal phospho-tyrosine domain: p.E573K, p.A599S, and p.F602L (Supplemental Figure 1C and Table 1). *SHP2* (also known as *PTPN11*) contained 3 previously unreported or rare heterozygous de novo or unphased missense variants, including the N-terminal SH2 binding-domain variant p.N58K and adjacent residue variants p.N308D and p.I309V in the C-terminal tyrosine phosphatase domain (Supplemental Figure 1D and Table 1). These results suggest that rare, damaging variants in a GRB2 signaling network including the upstream RTK receptor CSF1R (9) and downstream partner SHP2 (16–19) may confer risk for CH (Figure 1F).

### Co-expression of GRB2 and CSF1R in human microglia.

To explore GRB2 biology during human fetal brain development, we studied expression of GRB2 and CSF1R using a single-cell RNA-Seq atlas of 228,047 cells from early developing human brain spanning gestational weeks 5–23, as well as an atlas of 80,582 nuclei from human prenatal germinal matrix covering gestational weeks 18–38 (Figure 2A and Supplemental Figure 2A) (29, 30). Both *GRB2* and *CSF1R* transcripts were most highly differentially expressed in microglia as compared with other cell types (log_2_ fold change [fc] = 1.84, *P* = 1.0 × 10^–293^; log_2_fc = 2.96, *P* = 2.2 × 10^–308^, respectively (Figure 2, B and C), (log_2_fc = 1.04, *P* = 2.2 × 10^–308^; log_2_fc = 3.67, *P* = 2.2 × 10^–308^, respectively) (Supplemental Figure 2C). Co-expression of *GRB2* and *CSF1R* in both datasets was restricted almost exclusively to microglia, with *GRB2* expression peaking between human gestational weeks 21 and 23 (Figure 2D and Supplemental Figure 2D).

We also studied *Grb2* expression in an embryonic mouse dataset (31), which showed broad expression across all cell types, including microglia (Supplemental Figure 2E). Examination of *GRB2* expression in an adult human dataset (32) revealed similarly broad expression across multiple cell types (Supplemental Figure 2F).

The abundant expression of GRB2 in microglia prompted us to evaluate its expression in a dataset comprising 12,446 subclustered human microglial single-cell RNA-Seq transcriptomes spanning gestational weeks 5–23, labeled by inferred biological function (33). Among microglial subpopulations, *GRB2* was most highly differentially expressed in a cell subset classified as “endoplasmic reticulum stress” microglia (Supplemental Figure 3), which previously has been implicated in both neurodevelopment (34, 35) and neurodegenerative disease (36).

Consistent with this finding, enrichment analysis of the top differentially expressed genes in this cluster revealed the Gene Ontology (GO) biological process term “synaptic pruning” (GO:0098883; *P* = 2.4 × 10^–3^) to be the most enriched term, exceeded only by “response to endoplasmic reticulum stress” (GO:0034976; *P* = 1.8 × 10^–3^) (Figure 2E). Cell-cell communication analysis revealed significant signaling from *GRB2^+^ CSF1R^+^* microglial cells to radial glial, neural progenitor, and neuronal cell populations via the neural cell adhesion molecule, semaphorin-4A, and neuregulin pathways (Figure 2F).

Given the co-expression of *GRB2* and *CSF1R* in microglial cells, we sought to experimentally validate GRB2-CSF1R binding activities. IP revealed binding of GRB2 to CSF1R in mouse microglial cells as well as in human THP-1 monocytes (Figure 2G and Supplemental Figure 4). These data implicate a role for microglial GRB2 signaling in developing human brain and suggest that reduced quantities and/or function of GRB2 may contribute to disease.

### Grb2 deletion in mouse microglia phenocopies the hydrocephalus seen in patients with GRB2 mutation.

Human *CSF1R* variants are associated with hereditary diffuse leukoencephalopathy, a condition characterized by adult-onset, rapidly progressive neurodegeneration, and degenerative encephalopathy (12, 13), as well as with congenital brain malformations such as ventriculomegaly, agenesis of the corpus callosum, and Dandy-Walker malformation (14). Microglial-specific deletion of *Csf1r* in mice causes severe early postnatal hydrocephalus, accompanied by cerebral cortical thinning and a severely reduced number of brain microglia (10). Viewed together with our identification of patients with *GRB2*-mutant CH and the specific, overlapped expression of *CSF1R* and *GRB2* in human microglia (Figure 2, B and C), these data implicate a role for GRB2-expressing microglia in brain development.

To address this question, we generated a model of mononuclear phagocyte-specific *Grb2* KO (*Cx3cr1-Grb2^fl/fl^*) mice by crossing chemokine (C-X3-C motif) receptor 1 (*Cx3cr1)-Cre* with a conditional *Grb2* allele (Supplemental Figure 5A). Upon Cre-mediated recombination, exon 2 of *Grb2* is excised, leading to disruption of the N-terminal SH3 domain of GRB2 (Supplemental Figure 5A).

Cre-mediated recombination in the offspring eliminated or markedly reduced GRB2 protein expression in all monocytes, macrophages, and microglia (Supplemental Figure 5, A and B). Strikingly, live brain MRI showed severe, early postnatal CH and associated macrocephaly in approximately 50% of *Cx3cr1-Grb2^fl/fl^* mice (Figure 3, A–C). Hydrocephalic *Cx3cr1-Grb2^fl/fl^* mice had uncoordinated gait and failure to thrive, with a median survival of 28 days (Figure 3, A and D). Other *Cx3cr1-Grb2^fl/fl^* mice had mild-to-moderate ventriculomegaly. These findings indicate that *Grb2* deletion in microglia can reproduce the incomplete penetrance and full spectrum of ventriculomegaly, including CH, observed in patients with *GRB2*-mutant CH.

### GRB2-dependent hydrocephalus is associated with reductions in both cortical neuron mass and microglia during perinatal brain development.

CH can be either the communicating type (without intraventricular obstruction) or the obstructive type (with unambiguous intraventricular obstruction), most often caused by cerebral aqueduct stenosis. MRI studies of hydrocephalic *Cx3cr1-Grb2^fl/fl^* mice revealed cerebral aqueductal patency, indicating communicating hydrocephalus (Figure 3E) consistent with the phenotype of human patients with the *GRB2* mutation (Figure 1A). Impaired extraventricular CSF flow through pathways such as the glymphatic and meningeal lymphatic systems has recently been linked to communicating hydrocephalus (37) and to CNS immune homeostasis (38–42). Therefore, we assessed glymphatic function by slowly infusing artificial CSF containing fluorescent tracers of 2 different sizes into the cisterna magna of *Grb2^fl/fl^* and hydrocephalic *Cx3cr1-Grb2^fl/fl^* mice (43). Hydrocephalic *Cx3cr1-Grb2^fl/fl^* mice had significantly decreased tracer influx into the subarachnoid and perivascular spaces compared with controls, indicating reduced glymphatic-mediated CSF egress (Figure 3, F and G). These data demonstrate an association between GRB2-dependent communicating hydrocephalus and impaired glymphatic flux, either as a primary cause of CSF buildup or secondary to increased intracranial pressure.

Whereas acquired hydrocephalus is often linked to impaired CSF homeostasis (44), subsets of CH cases are associated with gene mutations that disrupt fetal neurogenesis and lead to significant ventriculomegaly by depleting neuronal cell mass in the cortical mantle (6, 8, 45–48). Therefore, we investigated the effect of conditional microglial Grb2 KO on cellular structure of developing cerebral cortex by immunostaining with Abs directed against Iba1, a microglia- and macrophage-specific cytoplasmic Ca^2+^-binding protein, and Abs against the neuron-specific RNA-binding protein NeuN. We observed a striking absence of Iba1^+^ microglia at P0, P7, P14, and P21 in the cortex of severely hydrocephalic *Cx3cr1-Grb2^fl/fl^* mice (Figure 4, A and B).

We also found that the microglial marker P2yr12 (49) was profoundly depleted in *Grb2*-mutant mice (Supplemental Figure 5A), whereas Olig2^+^ oligodendrocyte numbers were unaffected (Supplemental Figure 6B). Interestingly, Iba1^+^ cells were still present in non-hydrocephalic and mildly hydrocephalic *Cx3cr1-Grb2^fl/fl^* mice (Figure 4C). Hydrocephalic *Cx3cr1-Grb2^fl/fl^* mice also had significantly fewer NeuN^+^ cortical neurons (Figure 4, D and E). Immunolocalization of cortical layer markers Brn2, Ctip2, and Tbr1 (50–52) revealed that although cortical lamination was unaffected, the numbers of Brn2^+^ upper layer neurons and Tbr1^+^ deep-layer neurons were reduced in *Cx3cr1-Grb2^fl/fl^* mice (Supplemental Figure 6C). These findings demonstrate that Grb2 is essential for microglial development and suggest that ventriculomegaly results, in part, from microglia-dependent reduction in cerebral cortical neurons, as documented in *Csf1r*-mutant mice and humans with *CSF1R* mutation (10, 12–14).

### Unbiased proteomics reveals altered neurodevelopmental processes in GRB2-dependent hydrocephalus.

Last, we sought to understand the effects of microglia-specific *Grb2* KO on brain molecular architecture by performing unbiased mass spectrometry proteomics in WT and *Cx3cr1-Grb2^fl/fl^* mouse brains. Principal component analysis showed clear separation between WT and microglial-specific *Grb2* KO samples (Figure 5A). Similar to the immunostaining results, we found that P2RY12 was the most significantly decreased protein in *Grb2-*mutant brains compared with those of WT brains (Figure 5B). GO enrichment analysis of differentially expressed proteins showed changes in neurodevelopmental pathways in *Grb2*-mutant brains, including upregulation of synaptic signaling and neuron development, whereas gliogenesis, myelination, and neurogenesis were downregulated (Figure 5, C and D). Together, these results further suggest that Grb2 KO impairs microglial function, leading to disruptions of neuronal development and cortical neurogenesis. These findings mirror observations of depleted microglia and cortical thinning in *Csf1r* KO hydrocephalic mice (10) and suggest a paradigm in which brain microglia are essential for brain morphogenesis and CSF homeostasis.

## Discussion

In this study, we have identified a link among microglial GRB2, brain development, CSF homeostasis, and CH. Using human genetic data, developmental transcriptomic analyses, biochemical interaction studies, and a microglia-specific conditional KO mouse model, we show that disruption of GRB2 signaling leads to microglia loss, impaired cortical development, and communicating hydrocephalus. Biochemical experiments demonstrate GRB2-CSF1R binding interactions in mouse microglial cells and human monocytes. Together, these findings identify microglial GRB2 signaling as a previously unrecognized regulator of brain development and CSF homeostasis.

GRB2 is an adapter protein that acts as a linker between RTKs such as CSF1R and EGFR (53) and downstream Ras signaling pathway proteins such as SHP2 (Figure 1J). The SH2 domain of GRB2 binds to tyrosine-phosphorylated sequences in CSF1R, and its 2 SH3 domains facilitate complex formation with proline-rich regions of SHP2. Our biochemical studies validated the binding of GRB2 to CSF1R in mouse microglial cells and human monocytes. Mutations in CSF1R in both humans and mice mimic the ventriculomegaly seen in human patients and mice with *GRB2* mutation (10, 12–14). Loss-of-function heterozygous variants in CSF1R cause autosomal dominant, hereditary, diffuse leukoencephalopathy with spheroids (Online Mendelian Inheritance in Man [OMIM] 221820), while damaging compound heterozygous variants in CSF1R, leading to autosomal recessive brain abnormalities, neurodegeneration, and dysosteosclerosis (OMIM 618476), both characterized by severe ventriculomegaly or CH. Additionally, heterozygous variants in the SHP2-encoding gene *PTPN11* are associated with ventriculomegaly or CH in both LEOPARD syndrome 1 (OMIM 151100) (54, 55) and Noonan syndrome 1 (OMIM 163950) (23–25, 56). *Shp2*-mutant mice also display severe early postnatal hydrocephalus (20). Notably, we identified other patients with CH who had rare, damaging, or loss-of-function variants in CSF1R and SHP2 that map to domains interacting with GRB2. Together, these findings support a model in which disruption of the CSF1R/GRB2 signaling axis contributes to cerebral ventriculogenesis and CSF homeostasis (see Supplemental Table 1 for a summary of mouse and human evidence linking CSF1R and PTPN11/SHP2 to hydrocephalus and/or ventriculomegaly).

We used AlphaFold3 (AF3) multimer modeling (57) to explore potential interactions between GRB2 and its partners, including CSF1R and SHP2. Our *GRB2* variants are predicted to disrupt CSF1-stimulated CSF1R signaling by abrogating GRB2’s interaction with SHP2 via its SH3 domain. However, it is important to recognize that these are static models, and they do not account for post-translational modifications, conformational dynamics, or transient binding states. As such, although our structural predictions regarding the effects of the mutants are plausible, they remain speculative in the absence of experimental validation and should be interpreted with caution. Our AF3 prediction suggests an interaction between GRB2’s C-terminal SH3 domain and residues 562–568 of SHP2. This region lies in the C-terminal tail of SHP2, which is known to undergo tyrosine phosphorylation and interact with the GRB2 SH2 domain in a phosphorylation-dependent manner (58). This predicted binding site contrasts with previously reported proline-rich and SH3-binding motifs located within SHP2’s protein tyrosine phosphatase domain, considered canonical ligands of GRB2 SH3 domains (16). This discrepancy likely reflects alternative or context-dependent interaction modes and emphasizes the need for biochemical or biophysical studies to validate precise interaction interfaces. An additional limitation is that human CH-associated gene mutations may have gain-of-function effects; hence, the loss-of-function model we used in the mouse may not fully recapitulate the functional disruptions in humans. Nevertheless, we were able to confirm GRB2-CSF1R binding in mouse microglial cells and human monocytes through biochemical IP studies, and the loss-of-function mouse model exhibits hydrocephalus similar to what is seen in human patients. Future investigations making use of gene-edited cell lines could further characterize biochemical effects of human CH-associated GRB2 mutations.

Moreover, functional consequences of GRB2 mutations may arise not only from disrupted binding at interaction sites. Rather, they may reflect broader changes in the protein’s intramolecular energy landscape or allosteric regulation (59, 60). GRB2 functions as a modular adaptor with conformational flexibility and domain-domain interactions that mediate signal transduction (61, 62). Mutations could destabilize these regulatory networks or alter dynamic equilibria without directly abolishing binding to SHP2 or other partners, consistent with growing evidence for dynamic allosteric conformational transitions of SH3 domains, which regulate signaling output (16). Isolated domain behavior, however, may not fully recapitulate the dynamics and regulatory complexity of full-length GRB2. In light of these considerations, our structural predictions, though informative, are not definitive. The models, nevertheless, help in understanding the mutational impacts, particularly in the absence of structural data.

The co-expression of GRB2 with CSF1R in human microglia, along with the severe hydrocephalus and lack of microglia caused by deleting either gene, suggests that mid-gestational microglia are a key site of CSF1R-GRB2 activity in the developing brain. These findings also suggest that microglial dysfunction during critical periods of corticogenesis contributes to hydrocephalus, further supporting evidence that CSF1-regulated microglia functions are crucial in brain development (63). For example, microglia release cytokines (namely, IL-1β, IL-6, TNF-α, and IFN-γ) and signaling molecules (nitric oxide) that promote neurogenesis and control neuronal precursor numbers via phagocytosis (64). Similarly, during embryogenesis, microglia support proliferation of neuronal precursor cells, cortical neuron migration and differentiation, oligodendrogenesis, and astrogenesis (65, 66). However, single-cell transcriptomic atlases indicate expression of GRB2 in cell types other than microglia in human and mouse brain. Therefore, we cannot dismiss the possibility that human CH-associated GRB2 mutations may affect other cell types and act through different pathways, such as EGFR signaling (67). The finding that microglia-specific Grb2 KO is sufficient to cause hydrocephalus strongly indicates that microglia are key cellular contributors to the condition. Future research could uncover GRB2’s role in different cell types related to the hydrocephalus phenotype, using targeted genetic depletion methods and, potentially, patient-derived induced pluripotent stem cell models.

These data align with growing genetic evidence suggesting impaired neurogenesis may underlie or contribute to many cases of CH (6, 7, 48, 68). Our findings regarding GRB2 (and CSF1R) further support a “neural stem cell” model of hydrocephalus (6, 8, 45). In patients with *GRB2* variants, disrupted CSF1R-dependent microglial signaling could lead to reduced neural stem cell proliferation and cortical hypoplasia, with changes in biomechanical properties of the brain tissue contributing to hydrocephalus due to secondary CSF pooling in morphologically altered ventricles, similar to that caused by variants of the mRNA regulator TRIM71 (6, 69). An alternative, yet not mutually exclusive, hypothesis suggests CSF1R-GRB2 microglial signaling is essential to maintain cortical neurons after birth. Consistent with this hypothesis, microglia-derived IGF-1 is essential for neuron survival during early postnatal development, especially for layer V cortical neurons, which undergo apoptosis during temporary microglial depletion (70). Patients with *CSF1R* mutation and ventriculomegaly also show evidence of postnatal cortical neurodegeneration (71, 72).

These cases highlight the ambiguous boundary between congenital ventriculomegaly and hydrocephalus. Ventriculomegaly caused by the same gene variants has been treated with shunting in some patients but not others (46). For patients with the *GRB2* and *CSF1R* mutations, ventriculomegaly may result from impaired CSF homeostasis due to decreased glymphatic flux and reduced neuronal mass, as seen in disorders of neurogenesis or in neurodegeneration (6). *Csf1r* KO mice have increased numbers of astrocytes with fewer oligodendrocytes, consistent with glial scarring (10). Hydrocephalus in this context may arise from microglia-induced disruption in astroglial development, with glial scarring narrowing perivascular spaces and reducing CSF outflow. Without microglia, CSF pathways could be blocked by cell debris that microglia usually clear. These mechanisms may explain the reduced glymphatic CSF flux in our *Grb2* KO mouse. Future studies will clarify these processes.

Given the diversity of CH genetic causes and underlying biochemical pathways, these and other data might continue to support routine clinical WES for patients with newly diagnosed ventriculomegaly. Current guidelines for investigating fetal and neonatal ventriculomegaly include rapid commercial microarray testing for known chromosomal and copy-number abnormalities. However, this approach does not address CH cases explained by recently identified variants in many new CH risk genes. Using routine WES or whole-genome sequencing could improve treatment of children with CH by aiding in prognosis and treatment planning, increasing awareness of variant-related conditions through medical screening, and providing better counseling about recurrence risks, to boost reproductive confidence.

Important questions remain. For example, we cannot exclude possible dominant-negative or neomorphic effects of the described human GRB2 variants. However, in silico biophysical predictions suggest a deleterious impact on GRB2 function, and a conditional KO model of Grb2 recapitulated the human CH phenotype. Our results suggest incomplete penetrance and variable expressivity for *GRB2* variants, a phenomenon well recognized for other CH and NDD genes (8, 45). The determinants of incomplete penetrance and variable expressivity in the context of *GRB2* sequence variation remain unclear but could include common genetic or environmental modifiers (73, 74), as well as stochastic components resulting in mosaicism (75). Whole-genome sequencing focused on regulatory variants (e.g., enhancers and promoters adjacent to *GRB2* coding regions) and other genes within GRB2 biological pathways might help identify genetic modifiers.

To investigate the potential impact on CSF physiology, we performed cisterna magna injections of high-molecular-weight tracers, which permitted characterization of glymphatic drainage (76). A limitation of this approach is that CSF is 99% water (a molecular mass of only 18 Da). Nevertheless, the physiological roles of CSF include clearance of larger molecules, and impaired glymphatic function is a known contributor to hydrocephalus (77, 78). Our observation of decreased tracer clearance in Grb2-mutant mice, along with severe CV, strongly suggests disrupted CSF dynamics. Although direct measurement of fluid transport would be ideal, current techniques lack temporal and spatial resolution. Hence, tracer experiments remain the gold-standard method for assessment of CSF physiology (76). Although the precise molecular consequences of individual GRB2 variants remain to be fully defined, our genetic, biochemical, and in vivo findings establish an essential role of microglial GRB2 in brain development and identify disruption of this pathway as a candidate mechanism contributing to CH.

## Methods

### Sex as a biological variable.

Sex was not considered a biological variable in this study.

### Cohort of patients with CH.

Inclusion criteria included patients with primary CH or CV without prior genetic diagnosis. Excluded were patients with known chromosomal abnormalities, copy number variations, hydrocephalus caused by hemorrhage, infection, trauma, or CV due to obstruction by tumor, cyst, stroke, or posterior fossa crowding, as in Chiari II malformation. After consent, probands and family members provided buccal swabs, medical records, neuroimaging, operative reports, and phenotype data. Our cohort included 482 neurosurgically treated probands with CH.

### Exome sequencing and analysis.

Yale–Harvard cohort patient genomic DNA samples derived from saliva or blood were applied for exon capture using a Roche SeqCap EZ MedExome target enrichment kit or IDT xGen target capture followed by 101 or 148 base-paired-end sequencing on Illumina platforms, as described previously (79–81). Burrows-Wheeler Aligner-Maximal Exact Match was applied to align sequence reads to the human reference genome GRCh37/hg19. Single-nucleotide variants and small indels were called using a combination of the Genome Analysis Toolkit (GATK) HaplotypeCaller and FreeBayes (82) and annotated using ANNOVAR (83). Allele frequencies were annotated in the Exome Aggregation Consortium (ExAC) and the Genome Aggregation Database (GnomAD), version 2.1.1 (84). Variant filtration and analysis were conducted in accordance with GATK best practices and consensus workflows (85). Analyses were conducted separately for each class of variant (e.g., homozygous, heterozygous, de novo variants (DNVs) following previously established analytical methodologies. DNVs from the Harvard–Yale cohort were called from all CH parent-offspring trios using the established TrioDeNovo pipeline (86). GeneDx variants were called as previously defined (69, 87). Additional variants in *GRB2*, *CSF1R*, and *PTPN11* (SHP2) were identified from the GMKF database from original variant call files, where possible. Select phenotypic information was also ascertained from GMKF probands where possible, in concordance with GMKF policies to protect participant anonymity (88). Browser Extensible Data (BED) files of GRCh38/hg38 GMKF variant information were created using BEDtools, version 2.31.1 (89), and converted to GRCh37/hg19 using UCSC Genome Browser Database LiftOver (90) to maintain consistency with other datasets. Candidate variants for all samples were further filtered based on whether the variants were called in the exonic or splice-site regions, the variant read depth was at least 10 in the proband as well as both parents, and the global minor allele frequency was not greater than 4 × 10^−4^ in the ExAC and GnomAD. Samples from the Yale–Massachusetts General Hospital cohort were subsequently filtered based on the following criteria: (a) the proband’s alternative read depth was at least 5; (b) proband alternative allele ratio was at least 28% if there were fewer than 10 alternative reads, or not greater than 20% if there were at least 10 alternative reads; (c) the alternative allele ratio in both parents was not greater than 3.5%.

Samples from the GeneDx cohort were additionally filtered based on the following criteria: (a) genotype quality > 40 for all family members; (b) variant quality score log odds (VQSLOD) > –10; (c) Phred-scaled *P* value (Fisher’s exact test) <30; (d) proband alternate allele (ALT) count >4; (e) proband ALT ratio >0.1; (f) proband ALT ratio >0.15 if the reference allele (REF) and ALT calls are of equal length; (g) proband ALT ratio >0.25 if REF and ALT calls are of unequal length; (h) proband ALT ratio <0.9 in proband if DNV is autosomal; (i) DNV must be <100 bps for both the REF and ALT calls; (j) if the VQSLOD <7 and the ALT ratio in the proband is <0.3, the variant was omitted; And (k) DNVs were omitted if they existed in more than 2 unrelated probands. After filtering, in silico visualization was performed. We applied in-house software to manually inspect each variant for false-positive calls. Variants found to be false-positive upon manual inspection were removed. Variant annotations were then confirmed through manual cross-reference in the UCSC Genome Browser (90). Reported variants passing these filters and manual inspection were further confirmed by Sanger sequencing. A battery of predictive algorithms subsequently was applied to better understand variant impact on protein function, including combined annotation dependent depletion (CADD), version 1.6 (91), and meta-analytic support vector machine (MetaSVM), version 1.0 (92). Variants that met other filtering criteria were considered deleterious to protein function if the variants were of a class predictive to cause loss-of-function (stop-gain, start-loss, canonical splice site, and frameshift variants), as well as missense variants with CADD scaled score >20 or MetaSVM = damaging.

### Protein-protein interaction analysis.

The CSF1R-GRB2-PTPN11 multimeric complex was prepared using the AF3 server (Alphafoldserver.com). Briefly, AF3 uses an advanced deep-learning model to predict protein structures with high accuracy, including multimeric protein-protein interactions. The sequences of the human CSF1R (Uniprot identifier [ID] P07333; *n* = 972 aa), GRB2 (Uniprot ID P62993; *n* = 217 aa), and PTPN11 (tyrosine-protein phosphatase nonreceptor type 11; Uniprot ID Q06124; *n* = 593 aa) were obtained from the Uniprot database of protein sequence and functional information (www.uniprot.org). The sequences were then input to the AF3 server as 3 distinct proteins.

Initial trials at modeling the complex with complete sequences did not yield any meaningful results, because AF3 arbitrarily positioned the 3 different proteins in random orientations. CSF1R comprises 3 domains, namely the extracellular domain (residues 20–517), the transmembrane region (residues 518–538), and the intracellular domain (residues 539–972). CSF1R interacts with GRB2 at the intracellular side. Therefore, to force AF3 to model CSF1R interactions with GRB2 and PTPN11 via its intracellular domain, the first 520 aa corresponding to the CSF1R extracellular domain were removed, leaving CSF1R aa 521–972 to be modeled. Additional experiments from the literature suggested that phosphorylated Y697 was identified as a GRB2-binding site on CSF1R (9). Furthermore, the C-term SH3 domain (residues 156–215) in GRB2 interacts with the PTP domain (residues 247–517) in PTPN11 (16). The evidence for CSF1R interaction with PTPN11 was obtained from the human protein-protein interaction prediction server, yielding a favorable interaction score of 2.15 (93, 94). The disordered C-terminal region in PTPN11 (aa 573-593) with very low per-residue confidence score (predicted local distance difference test <50) was deleted from the model, leaving PTPN11 aa 1-573 to be modeled. This final AF3 model satisfied these previously published experimental findings. The mutations were then constructed and the free energy of change (ΔΔG) was calculated using the Internal Coordinate Mechanics mutagenesis program (www.molsoft.com). Structure figures were generated using Pymol-MDAnalysis exploratory project.

### Single-nuclei RNA-Seq analysis.

Processing of prenatal human germinal matrix, early developing human brain, and microglia datasets have been previously described (29, 30, 33). The prenatal human germinal matrix dataset was obtained directly from the authors, and the early human brain development and microglia datasets were obtained from the OMIX database of the China Genomics Data Center (accession numbers OMIX005402-02 and OMIX931-99-01). Downstream analysis was completed in Seurat (version 4.3.0) (95). Average expression of genes across gestational weeks was calculated using the AverageExpression command and differential expression was calculated using the Wilcoxon’s rank sum test by running the FindAllMarkers command. Enrichment analysis was performed on the top 100 differentially expressed genes identified by the FindAllMarkers function in the endoplasmic reticulum stress microglia subcluster using the EnrichR R package (version 3.2) (96). *P* values were calculated using Fisher’s exact test, and the Benjamini–Hochberg procedure was applied to correct for multiple hypotheses. Cell-cell communication analysis was performed using CellChat (version 2.1.2) following the standard analysis pipeline. The germinal matrix dataset was subset to gestational weeks 20–24, and the early fetal human brain dataset was subset to gestational weeks 20–23 for CellChat analysis. The functions netVisual_aggregate and netVisual_chord_gene were used to generate chord diagrams.

### Animals.

Mice were reared under the veterinary care provided by the Yale Animal Resource Center in standardized IACUC-approved husbandry and housing conditions. Groups of <5 mice per cage were housed at 25°C and 56% humidity on a 12-hour light/dark cycle. Animals were always provided food and water with no restrictions, and both sexes were used in experimentation. *Cx3cr1-Cre* (The Jackson Laboratory, strain 025524) were mated with conditional *Grb2^fl/fl^* mice (The Jackson Laboratory, strain 020422) to generate *Cx3cr1-Grb2^fl/fl^* mice, in which *GRB2* was functionally deleted from all macrophage-lineage cells.

### Animal MRI.

Mice were anesthetized with isoflurane in a medical air and oxygen mixture of 1:0.5 (3% induction, 1%–2% maintenance). Mice were then placed in and imaged on an 11.7T preclinical Bruker magnet using an in-house-built saddle coil (6). In 31 minutes and 30 seconds, using a repetition time/echo time of 4,500/11.25 ms, 20 repetitions, and Rapid Acquisition with Relaxation Enhancement factor 8, we obtained a coronal 0.07 × 0.30 × 0.07 mm^3^ image of the whole brain (field of view, 1.40 × 8.40 × 12.0 mm^3^). This sequence was repeated, and the data were averaged after motion correction using BioImage Suite (6). We then used the signal-intensity thresholding feature on BioImage Suite to delineate the ventricular system from the surrounding brain parenchyma and to calculate ventricular volume.

### Animal tissue preparation and immunostaining.

At indicated time points, mice were transcardially perfused with ice-cold PBS, followed by 4% paraformaldehyde (PFA) in PBS. Brains were dissected, placed in 4% PFA overnight at 4°C, and then cryoprotected in 30% sucrose in PBS for 48 hours. After this process, brains were placed in frozen OCT blocks, sectioned at 20 μm on a cryostat, and mounted on microscopy slides. Sections (mounted on slides) were stored at −80°C. When sections were needed for staining, we first removed the slides from the freezer and let them thaw at room temperature. Once thawed, slides were washed with 0.5% PBST and blocked in 10% normal goat serum (NGS) in PBST at room temperature for 1 hour. Slides were then incubated with primary Abs diluted in 2.5% NGS in PBST at 4°C overnight. After primary Ab incubation, sections were washed and incubated with Alexa Fluor–conjugated secondary Abs (1:500 in 2.5% NGS in PBST) for 1 hour at room temperature. Nuclei were counterstained with DAPI/PBST (5 ng/mL) for 10 minutes, washed 3 times with PBST for 5 minutes each, air-dried, and mounted on coverslips with Prolong Gold Antifade Mounting Medium. Images were acquired using a Zeiss LSM 800 confocal microscope. The following primary Abs (with dilutions) were used: goat anti-Iba1 (Abcam, ab2224402) and rabbit anti-NeuN (Abcam, ab177487). All secondary Abs were purchased from Invitrogen. The following were used: goat anti-rabbit IgG (H+L) Alexa Fluor 488 (A-11008), goat anti-rabbit IgG (H+L) Alexa Fluor 555 (A-21428), and donkey anti-goat IgG (H+L) Alexa Fluor 647 (A-21447).

### Cisterna magna injections and image analysis.

The day before the experiment, mice were anesthetized with 100 mg/kg ketamine and 10 mg/kg xylazine, administered intraperitoneally. A 30 gauge needle connected to closed-end PE10 tubing was inserted into the cisterna magna. The needle and tubing were secured with superglue and dental cement. All subsequent tracer experiments were performed in awake animals by injecting 5% Alexa647-conjugated BSA (66 kDa) or 5% Alexa647-conjugated dextran (10 kDa) in artificial cerebrospinal fluid into the cannula implanted into the cisterna magna the day before. Tracer was infused at a rate of 1 μL/min using the Harvard Instruments syringe pump (Series 11 Elite). The total circulation time of the CSF tracer was 30 minutes from the start of injection.

At the end of the experiment, mice were anesthetized with inhalable isoflurane (2.5% in 1 L oxygen/min) and rapidly decapitated. Brains were removed and fixed in 4% PFA in PBS overnight at 4°C. The next day, brains were sectioned, using a Leica vibratome, into 100 μm–thick coronal slices. Brain slices were imaged using a ×0.63 lens with ×2 magnification on an MvX10 microscope (Olympus) using a Lumencore 1600 light source (Prior) with Metamorph Basic software (Olympus). Images were quantified by FIJI (ImageJ, NIH). Each coronal section was outlined manually, and the percentage of area covered by tracer was determined using a previously verified threshold function (43, 97).

### Isolation of mouse microglia.

Primary microglial cultures were prepared from the cortices of P0–P2, male, C57BL/6 WT pups, using previously established protocols (98). Briefly, brain tissue was enzymatically dissociated with papain (Miltenyi Biotec), and the resulting cell suspension was maintained in T75 flasks using L929-conditioned medium. Microglia were cultured on an astrocyte feeder layer for 2 weeks before being isolated by mechanical agitation. After isolation, microglia were matured for 4–5 days in media supplemented with TGF-b1 (50 ng/mL) and macrophage-CSF (M-CSF) (10 ng/mL) prior to lysis.

### Immunoprecipitation.

We obtained 1000 μg of protein lysate from primary microglia (mouse) or unstimulated THP1 cells (human; ATC TIB-202) and combined that with 10 μL of a 0.2 mg/mL solution of rabbit monoclonal anti-rabbit Grb2 (clone 9K6H0; Invitrogen, Thermo Fisher Scientific, MA5-35238) per sample in a microcentrifuge tube. Using the Pierce Classic Magnetic IP/Co-IP Kit (Thermo Fisher Scientific, 88804) according to manufacturer’s instructions, the Ab/lysate solution was then diluted with IP lysis/wash buffer and incubated overnight at 4°C. The antigen sample/Ab mixture was incubated with prewashed magnetic beads for 1 hour with mixing, after which beads were magnetically collected and washed 3 times with IP lysis/wash buffer. Then, beads were mixed with ultrapure water and collected. After a 10-minute incubation in elution buffer, beads were magnetically separated, and the supernatant containing the target antigen underwent Western blot for CSF1R (Invitrogen, Thermo Fisher Scientific, PA5115557) according to accepted methods used by our group (99).

### Sample preparation for whole-brain proteomics.

Tissue in 160 μL of stock RIPA (containing protease [Pierce, 87786] at 1% of lysis buffer) and phosphatase inhibitor cocktails (Pierce, 78420) at 2.5% of lysis buffer) were sonicated, vortexed, and centrifuged. Then, 40 μL was aliquoted into a new tube, and proteins were reduced with DTT at 37°C for 30 minutes, cooled to room temperature, and alkylated with iodoacetamide in the dark at room temperature for 30 minutes. Chloroform:methanol:water protein precipitation was performed, and dried protein pellets were resuspended in 200 μL of 8 M urea containing 400 mM ammonium bicarbonate. After protein quantitation (Nanodrop), 50 μg of protein was transferred to a new tube. Proteins were digested overnight with shaking by LysC (2 μL at 0.5 μg/μL) at 37°C. Trypsin (2 μL of 0.5 μg/μL) was then added and the mixture incubated 6.5 hours with shaking. Digestion was quenched with 20% trifluoroacetic acid. Samples were desalted using a BioPureSPN PROTO 300 C18 macro spin columns (The Nest Group, HMM S18V) and dried via SpeedVac (Thermo Scientific, SAVANT RVT-4104). Peptide concentrations were determined by NanoDrop 2000 (Thermo Scientific). We took 0.3 μg/5 μL per sample for liquid chromatography–tandem mass spectrometry (LC MS/MS) analysis.

### LC-MS/MS data collection.

A Thermo Scientific Q-Exactive Plus Mass spectrometer was connected to a Waters nanoACQUITY UPLC system equipped with a Waters Symmetry C18 180 μm × 20 mm trap column and a 1.7-μm, 75 μm × 250 mm nanoACQUITY UPLC column (35°C). In block randomized order, 5 μL of each digest at a concentration of 0.05 μg/μL was injected. To ensure high-level identification and quantitation integrity, a resolution of 70,000 was used for MS. A total of 20 MS/MS spectra (17,500 resolution) were acquired per MS scan using higher-energy collisional dissociation. All MS (profile) and MS/MS (centroid) peaks were detected in the Orbitrap. Trapping was carried out for 3 minutes at 5 μL/min in 99% buffer A (0.1% formic acid in water) and 1% buffer B (0.075% FA in acetonitrile) prior to elution, with linear gradients reaching 5% B at 2 minutes, 25% B at 140 minutes, 40% B at 165 minutes, and 90% B at 170 minutes. This eluate was held at 90% B for 10 minutes longer prior to dropping the percentage of B back to 0.5% at 182 minutes. Three blanks (2 of 100% acetonitrile and 1 of buffer A) followed each sample injection to ensure against sample carryover. The MS quality control included Hela cell and transferrin digest injections.

### LC MS/MS Data Analyses.

LC MS/MS data were analyzed with Proteome Discoverer (PD), version 3.0 (Thermo Fisher Scientific), with the Mascot search engine (version 2.7 Matrix Science) (100), using the *Mus musculus* species protein database. Resulting PD analyses were imported into Scaffold (version 4.1, Proteome Software) for further interrogation and data filtration. Positive protein identifications were based on hits with 2 or more unique peptides per protein, and peptides were considered significant if the Mascot score was higher than the 95% confidence level.

The protein database search was conducted in-house using the Mascot algorithm (100) for uninterpreted MS/MS spectra. The data were searched against the SWISSPROT *Saccharomyces cerevisiae* protein database. The search parameters were as follows: type of search = MS/MS ion search; enzyme= LysC/trypsin; variable modifications = oxidation (Met), methylthio (C); mass values = monoisotopic; protein mass = unrestricted; peptide mass tolerance = ±10 ppm; fragment mass tolerance = ±0.02 Da; charge = 2+, 3+, 4+; maximum missed cleavages = 2; decoy = yes; and instrument type = ESI-TRAP.

### Statistics.

All statistical analyses were performed using GraphPad Prism, version 10.0.0 for Windows (GraphPad Software). Data for ventricular volume, cell counting, and tracer influx experiments were analyzed in a blinded manner. Statistical significance was determined using a 2-tailed Student’s *t* test. A *P* values of less than 0.05 was considered statistically significant.

### Study approval.

All study procedures complied with the Human Investigation Committee and Human Research Protection Program at Yale School of Medicine (New Haven, CT, USA) and Massachusetts General Hospital (Boston, MA, USA). All probands provided written informed consent in accordance with the Declaration of Helsinki. For pediatric patients, consent was also obtained from guardians. All experiments using mice were performed under a protocol previously approved by the IACUC of Yale University and in accordance with the guidelines outlined in the NIH Guide for the Care and Use of Laboratory Animals.

### Data availability.

Mouse proteomics data have been deposited to the PRIDE database (ProteomeXchange accession: PXD081055). All other data can be found in the “Supporting data values” file.

## Author contributions

KTK and ELH conceptualized the study, acquired funding, and served as project administrators and supervisors. KTK, ELH, HS, BCR, and PQD devised the study methodology. BCR, HS, GA, ED, NHM, PQD, SH, BF, KYM, LTH, MB, AJP, CNW, XP, PK, LW, DRR, SZ, QL, WCD, and SS conducted the investigation. BPK, PLM, SLA, SCJ, and TTL provided resources and methodological expertise. GA, NHM, SH, and BF contributed to data visualization. PQD, BCR, KTK, ED, and GA wrote the original draft of the manuscript, and all authors reviewed and edited the manuscript. Co–first author position was determined by the order in which those authors joined the project.

## Conflict of interest

KTK is a consultant for Aesculap Inc. and is a co-founder of Ventra Neuroscience Inc. EH is a consultant for Boehringer Ingelheim, Iqvia, Merck, and Bristol Myers. BPK is a consultant for Foresite Capital and Jumble Therapeutics and is a member of the scientific advisory boards of Life Edit Therapeutics and Prime Medicine. He also holds a financial interest in Prime Medicine, Inc., which is developing therapeutic CRISPR-Cas gene-editing technologies. BPK is an inventor on patents and patent applications filed by MGB that describe genome engineering technologies; a complete list of patent numbers is provided in supplemental material.

## Funding support

This work is the result of NIH funding, in whole or in part, and is subject to the NIH Public Access Policy. Through acceptance of this federal funding, the NIH has been given a right to make the work publicly available in PubMed Central.

- NIH (grants 1R01NS109358-01 and 1R01NS111029-01A1, to KTK).
- The Rudi Schulte 797 Organization (to KTK).
- The Hydrocephalus Association (to KTK).
- Simons Foundation (to KTK).
- March of Dimes (to KTK).
- Friedreich’s Ataxia Research Alliance (FARA) (to DRR).
- FARA Australia Postdoctoral Fellowship (to DRR).
- NIH grant PO1HL142494 (to BPK).
- NIH grant F32AG089892 (to PQD).
- Kayden-Lambert MGH Research Scholar Award (to BPK).

## Supplementary Material

Supplemental data

Unedited blot and gel images

Supporting data values

## Figures and Tables

**Figure 1:**
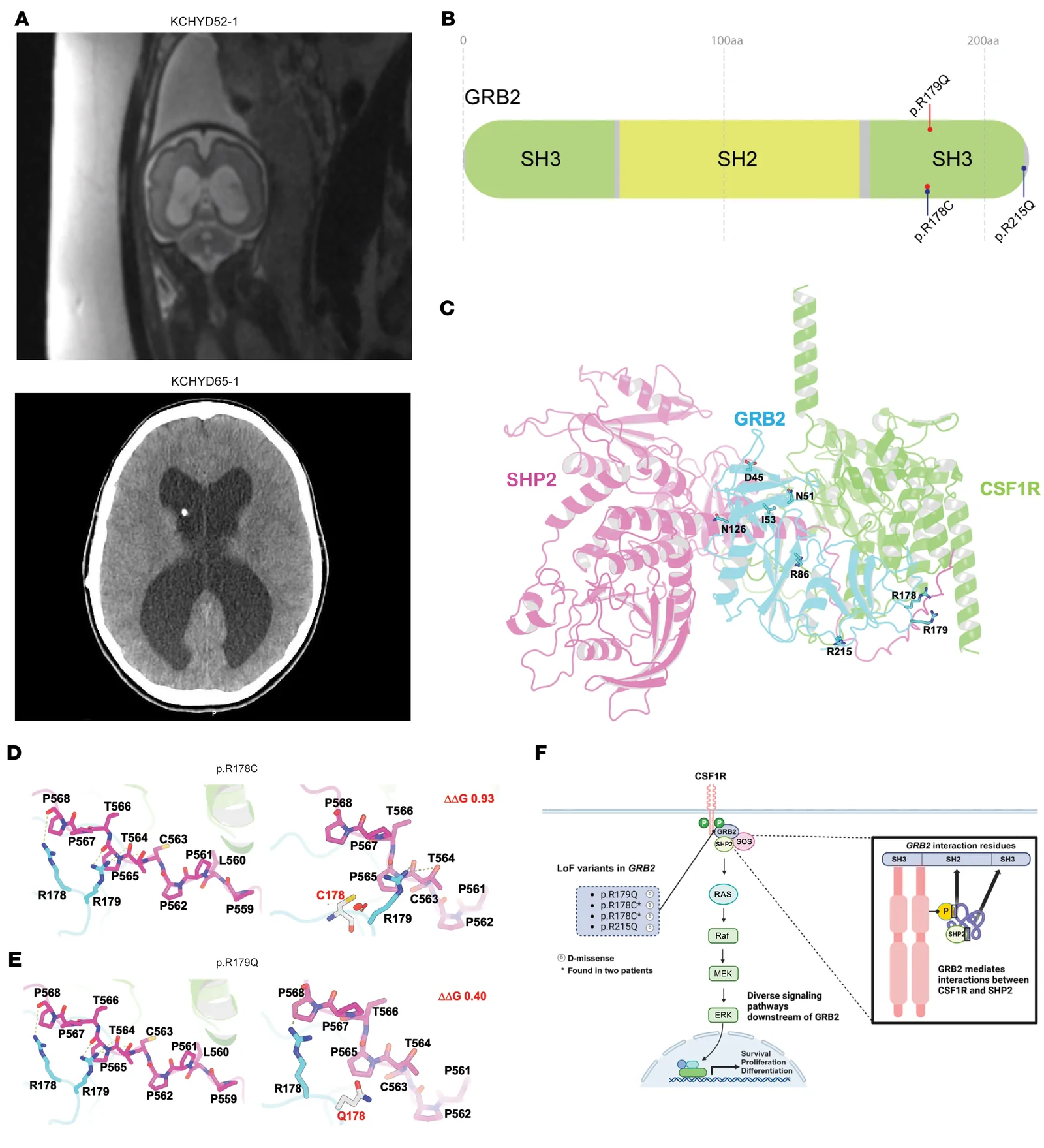
Recurrent, damaging variants in the GRB2 c-terminal SH3 domain in patients with CH. (**A**) Coronal T1-weighted fetal MRI of proband KCHYD52-1 and axial head CT scan of proband KCHYD65-1 (white dot indicates ventricular catheter of a ventriculo-peritoneal shunt used to treat hydrocephalus). (**B**) Schematic of variant locations in *GRB2* protein domains: red variants are from our CH cohort, blue variants are from the GMKF database. The axis depicts protein length (aa, in gray). (**C**) Final AF3 model of the CSF1R-GRB2-SHP2 multimeric complex, with sites of GRB2 missense substitutions highlighted. Shown are CSF1R aa 521–926, GRB2 aa 1–217 and SHP2 aa 1–573. AlphaFold models of deleterious missense variant p.R178C (ΔΔG 0.93) (**D**) and p.R179Q (ΔΔG 0.40) (**E**), both predicted to be detrimental to GRB2 structure and function due to loss of hydrogen-bonding interactions between GRB2-mutant side chains and adjacent SHP2 backbone atoms. (**F**) Schematic of the CSF1R/GRB2/SHP2 signaling pathway with all variants summarized. LoF, loss of function.

**Figure 2:**
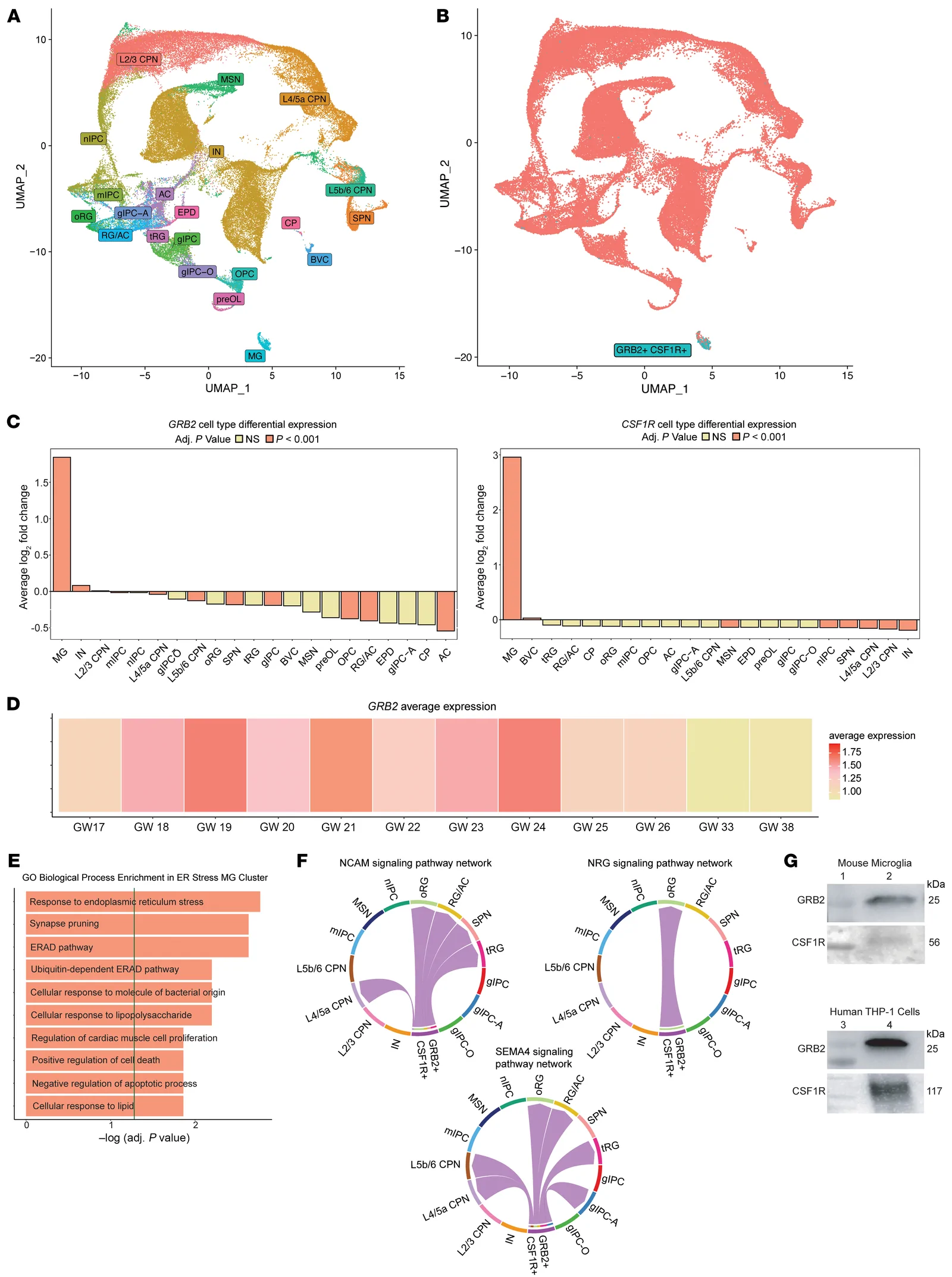
Expression of *GRB2* with *CSF1R* in a subset of microglia in developing human brain. (**A**) Uniform Manifold Approximation and Projection (UMAP) plot of prenatal human germinal matrix colored by main cell type. RG (radial glia), oRG (outer radial glia), tRG (truncated radial glia), EPD (ependymal cell), AC (astrocyte), gIPC (glial intermediate progenitor cell), OPC (oligodendrocyte progenitor cell), preOL (premyelinating/early myelinating BCAS1^+^ oligodendrocyte), OL (oligodendrocyte), nIPC (neuronal intermediate progenitor cell), mIPC (multipotent intermediate progenitor cell), MSN (medium spiny neuron), EN (excitatory neuron), CPN (cortical projection neuron), SPN (subplate neuron), IN (interneuron), MG (microglia), BVC (blood vessel cell), CP (choroid plexus), and L2/3, L4, L5/6 (neocortical layers 2/3, 4, 5/6). (**B**) UMAP plot identifying *GRB2^+^ CSF1R^+^* cells. (**C**) *GRB2* and *CSF1R* differential expression across cell types. (**D**) Temporal expression of *GRB2* between gestational weeks 17 and 38. (**E**) Enrichment analysis showing top GO Biological Processes enriched in the endoplasmic reticulum stress microglia subcluster. (**F**) Chord diagrams demonstrating signaling from *GRB2^+^ CSF1R^+^* cells to other cell populations in prenatal germinal matrix (gestational weeks 20–24). Neural cell adhesion molecule (NCAM), Semaphorin-4A (SEM4A), and neuregulin (NRG) pathway networks. (**G**) Total cell lysates prepared from primary mouse microglia and unstimulated human THP1 cells underwent GRB2 IP followed by Western blotting for CSF1R. In both cell types, Western blotting of the GRB2 IP product revealed a band corresponding to the full-length CSF1R (56 kDA in mouse, 117 kDA in human). Lanes 1 and 3: MW marker. Lane 2: primary mouse microglia. Lane 4: unstimulated THP1 cells.

**Figure 3:**
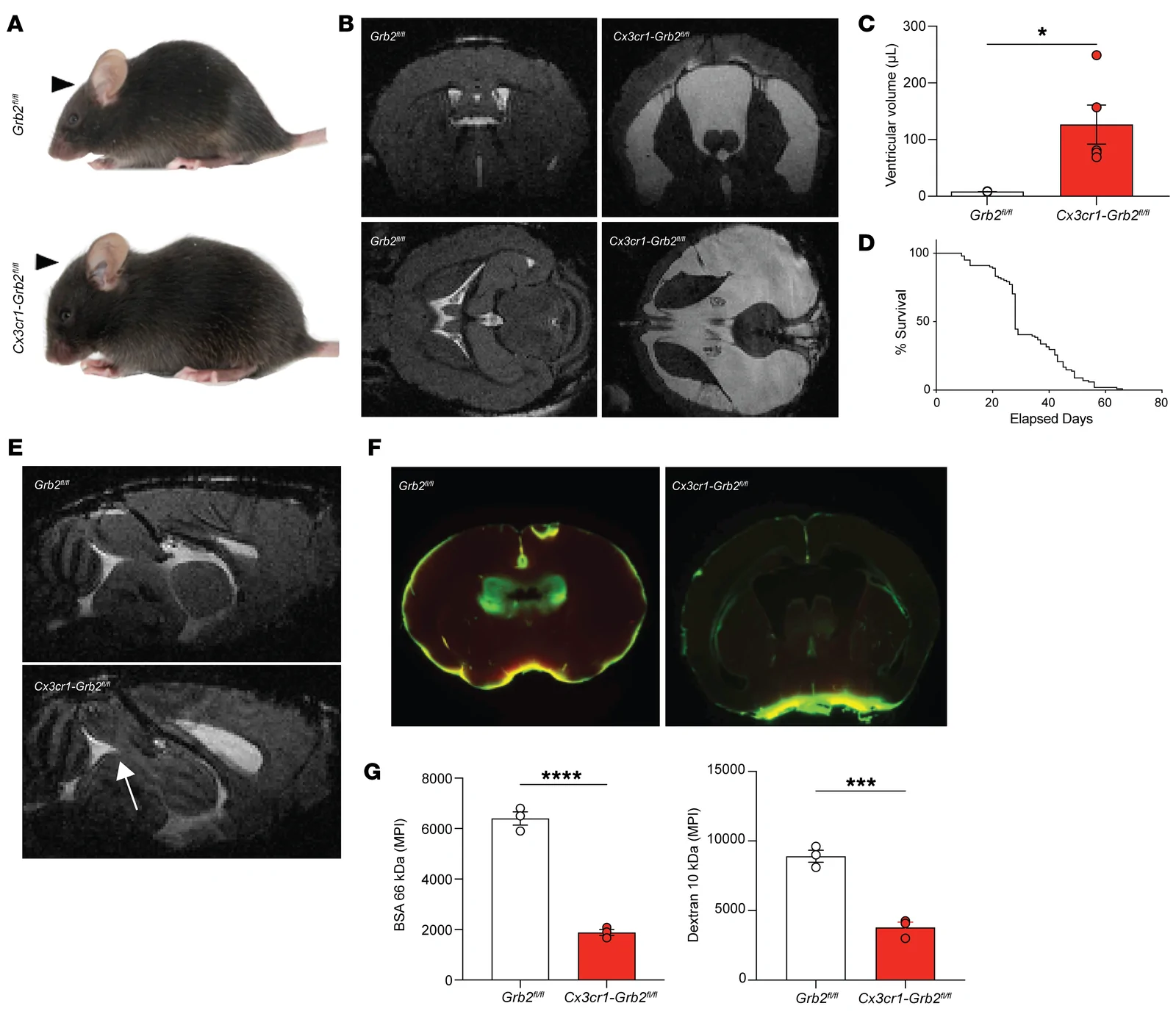
Microglial-specific KO of *GRB2* in mice results in severe CH characterized with impaired glymphatic flux. (**A**) Representative images of a phenotypically normal *Grb2^fl/fl^* mouse (top) and a macrocephalic *Cx3cr1-Grb2^fl/fl^* mouse (bottom) at P21. Black arrowheads illustrate bossing in the mutant mouse. (**B**) Representative coronal and axial MRI images showing severe ventriculomegaly in a *Cx3cr1-Grb2^fl/fl^* mouse (right) compared with a *Grb2^fl/fl^* control (left) at P30. (**C**) Quantitation of (**B**): ventricular volume from *Grb2^fl/fl^* controls and from a random selection of the approximately 40% of *Cx3cr1-Grb2^fl/fl^* mice that demonstrated severe ventriculomegaly at P30 (*n* = 3–5 animals per condition). Statistical significance was determined by Student’s *t* test (2-tailed). *P < 0.05. (**D**) Kaplan-Meier survival curve of hydrocephalic *Cx3cr1-Grb2^fl/fl^* mice (*n* = 107). (**E**) Representative sagittal MRI images from *Grb2^fl/fl^* control and hydrocephalic *Cx3cr1-Grb2^fl/fl^* mice. White arrow designates cerebral aqueduct. (**F**) Representative cross-sections after cisterna magna injection of BsA 66 kDa tracer into *Grb2^fl/fl^* control (left) and hydrocephalic *Cx3cr1-Grb2^fl/fl^* (right) mice. (**G**) Quantitation of (**F**): BsA 66 kDa and Dextran 10 kDa tracer accumulation in subarachnoid and perivascular spaces after cisterna magna injection (*n* = 3 mice per condition). Statistical significance was determined by Student’s *t* test (2-tailed). ****P < 0.0001, ***P < 0.001. MPI, mean pixel intensity.

**Figure 4:**
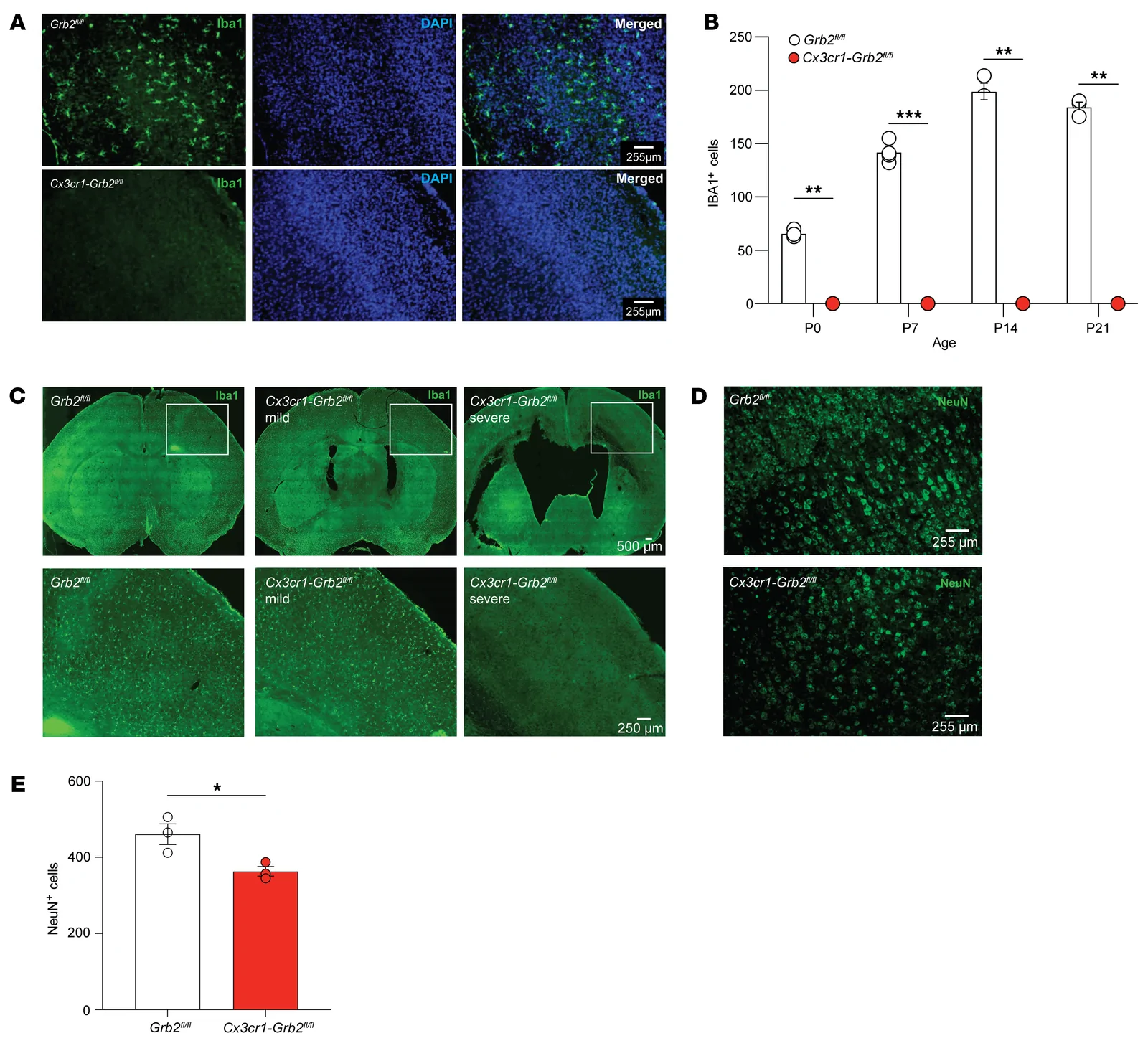
Depletion of microglia and reduced cortical neurogenesis in hydrocephalic *Grb2-*mutant mice. (**A**) Representative immunofluorescent (IF) images of Iba1 (green) and DAPI (blue) in P7 cortex of *Grb2^fl/fl^* control (top) and hydrocephalic *Cx3cr1-Grb2^fl/fl^* (bottom) mice. (**B**) Quantitation of (**A**) showing the number of Iba1^+^ cells counted in the cortex of *Grb2^fl/fl^* controls and of hydrocephalic *Cx3cr1-Grb2^fl/fl^* mice of indicated ages (*n* = 3–4 mice per condition; each data point represents the average Iba1^+^ cell number per cortical section, 3–4 sections per mouse). Statistical significance was determined by 2-way ANOVA test followed by Šidák’s multiple comparisons test. **P < 0.01, ***P < 0.001. (**C**) Representative IF images of IBA1 (green) in the P7 brains of a control *Grb2^fl/fl^* mouse, a *Cx3cr1-Grb2^fl/fl^* mouse with mild ventriculomegaly, and a *Cx3Cr1-Grb2^fl/fl^* mouse with severe ventriculomegaly. (**D**) Representative IF images of cortical NeuN in *Grb2^fl/fl^* control (top) and hydrocephalic *Cx3cr1-Grb2^fl/fl^* (bottom) mice. (**E**) Quantitation of (**D**) showing the number of cortical NeuN^+^ cells in *Cx3Cr1-Grb2^fl/fl^* control mice and in hydrocephalic *Cx3cr1-Grb2^fl/fl^* mice (*n* = 3 mice per condition; with each data point representing the average number of NeuN^+^ cells per cortical section, 3–4 per mouse). Statistical significance was determined by Student’s *t* test (2-tailed). *P < 0.05.

**Figure 5:**
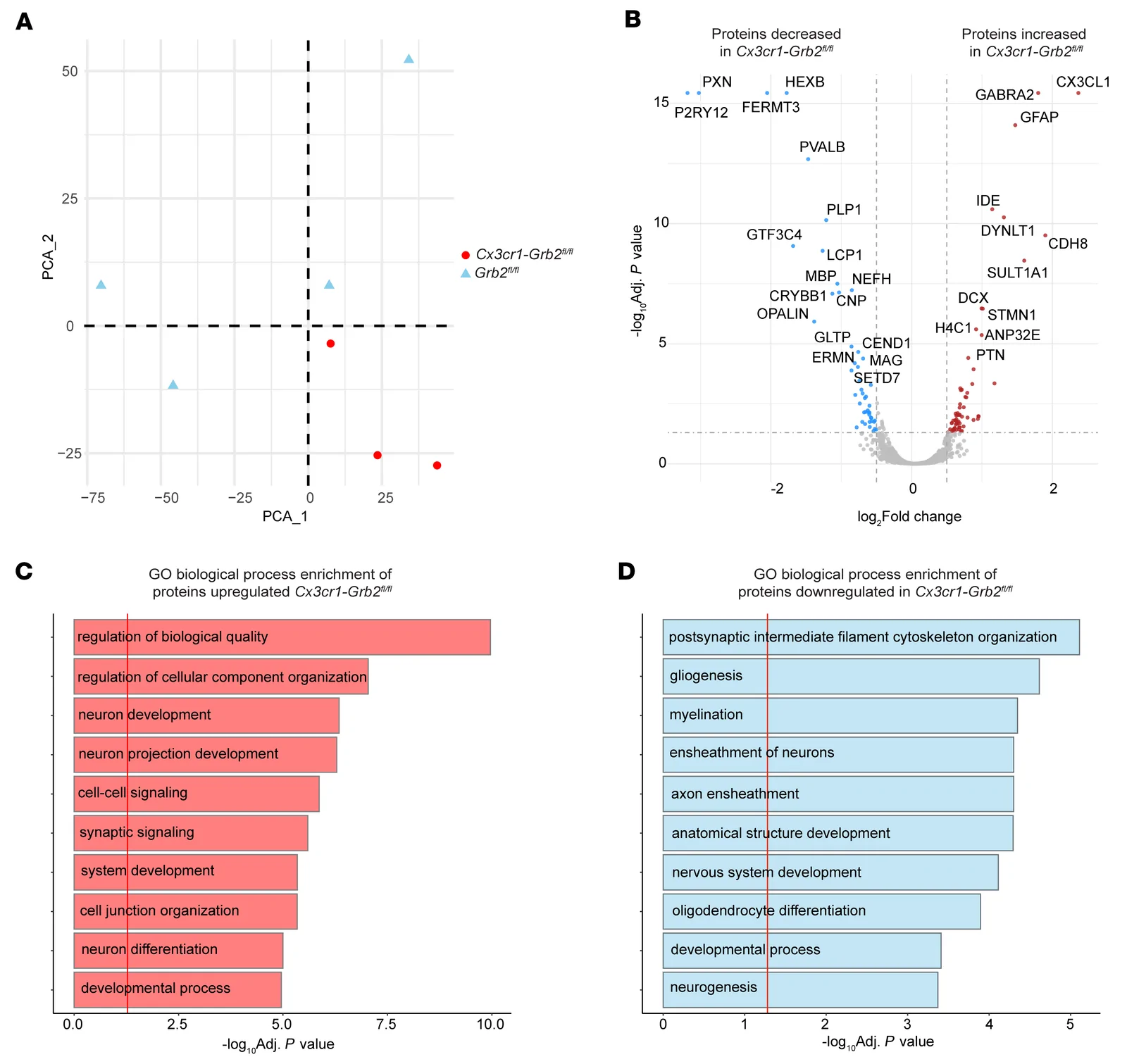
Unbiased proteomics reveals altered neurodevelopmental pathways in the hydrocephalic cortex of *Cx3cr1-Grb2^fl/fl^-*mutant mice. (**A**) Principal component analysis (PCA) of proteomic data from whole brains of *Grb2^fl/fl^* and hydrocephalic *Cx3cr1-Grb2^fl/fl^* mice. Each point represents a biological replicate. (**B**) Volcano plot comparing differential protein abundance in whole brains of *Grb2^fl/fl^* and *Cx3cr1-Grb2^fl/fl^* mice. (**C**) GO biological process pathway enrichment of significantly upregulated proteins in *Cx3cr1-Grb2^fl/fl^* mice mutant compared with *Grb2^fl/fl^* control mice. (**D**) GO biological process pathway enrichment of significantly downregulated proteins in *Cx3cr1-Grb2^fl/fl^* mice compared with *Grb2^fl/fl^* control mice.

**Table 1:**
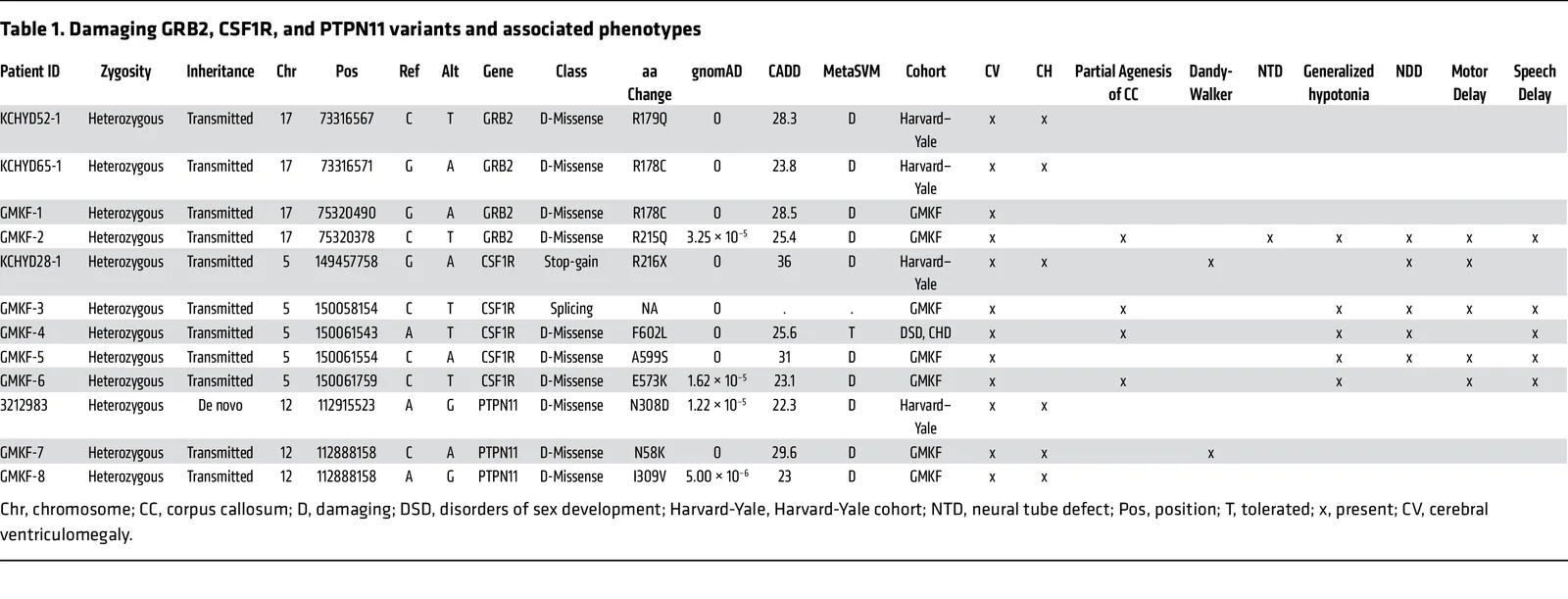
Damaging GRB2, CSF1R, and PTPN11 variants and associated phenotypes

## Version 1 09/10/2026

In-Press Preview

## Version 2 10/15/2026

Electronic publication
