# Supplementary material for: Microglial GRB2 is essential for brain ventriculogenesis and cerebrospinal fluid homeostasis
Source: J Clin Invest. 2026 Sep 10;136(20):e185791. doi: 10.1172/JCI185791 (PMC13668624; doi:10.1172/JCI185791)
Supplement: Supplementary file 1 [file jci-136-185791-s075.pdf]

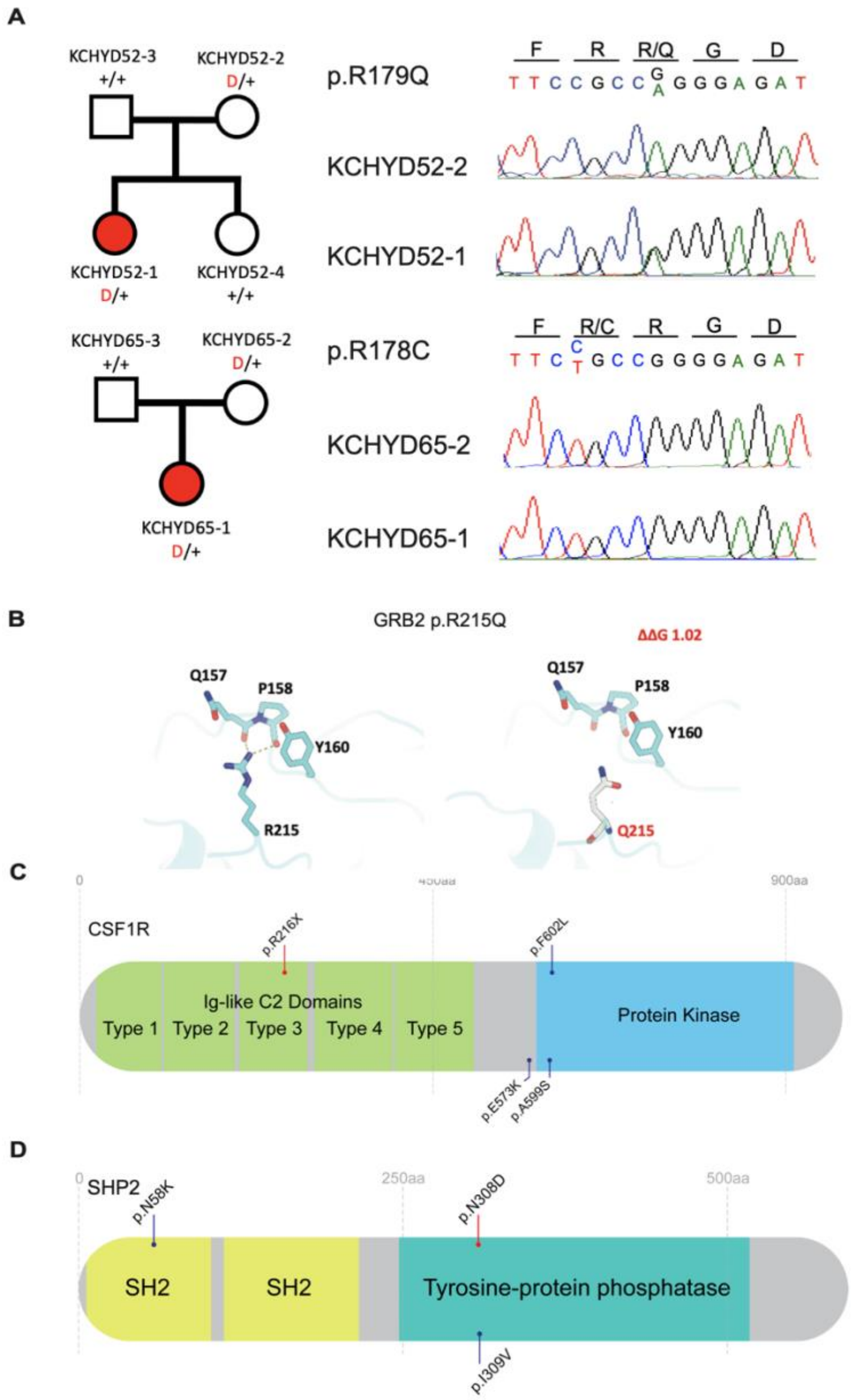

Supplementary Figure 1: Protein-protein interaction modeling of GRB2 variant p.R215Q. (A) Pedigrees and Sanger sequencing electropherograms depicting *GRB2* mutations in genomic DNA from congenital hydrocephalus proband KCHYD52-1 and KCHYD65-1. (B) Deleterious missense variant p.R215Q (DDG 1.02) is predicted by Alpha-Fold as detrimental to *GRB2* structure and function to loss of multiple hydrogen bonds that normally constrain loop region flexibility, thus indirectly destabilizing the adjacent constitutive GRB2 phosphorylation site Y160. (C) Schematic diagrams of CSF1R protein and (D) SHP2 protein domains showing variant locations. Variants from our CH cohort are indicated in red, and variants from GMKF database are in blue. Axis depicts protein length (aa, in gray).

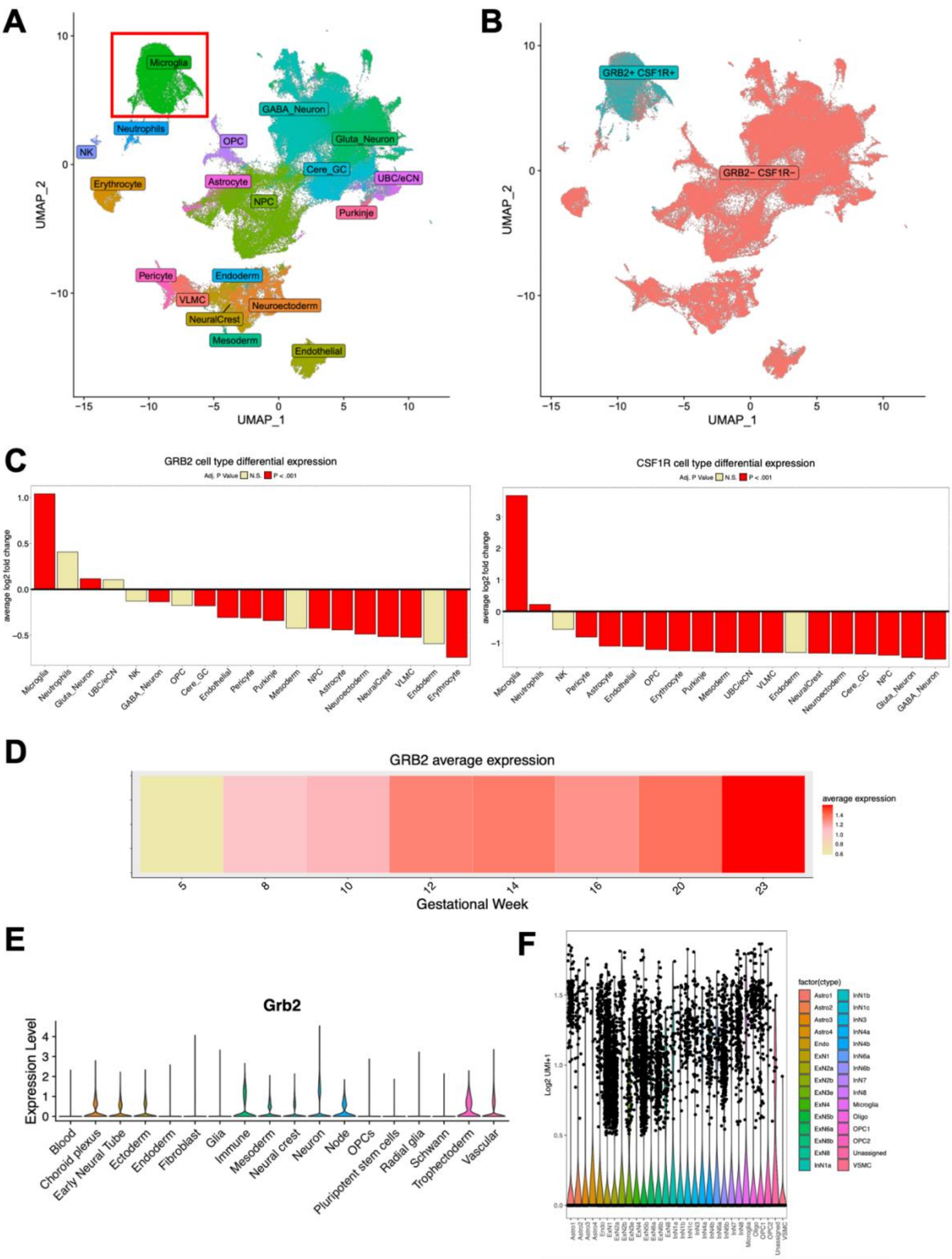

Supplementary Figure 2: *GRB2* and *CSF1R* expression profiling in a single-cell atlas of early developing human brain development. (A) UMAP plot colored by main cell type. Neural progenitor cell (NPC), common glutamatergic neuron (Gluta_Neuron), Cerebellar granule cell (Cere_GC), unipolar brush cell/excitatory cerebellar nuclei (UBC/eCN), common GABAnergic neuron (GABA_Neuron), oligodendrocyte precursor cell (OPC), and vascular leptomeningeal cells (VLMC). (B) UMAP plot identifying *GRB2+ CSF1R+* cells. (C) *GRB2* and *CSF1R* differential expression across cell types. (D) Temporal expression of *GRB2* between gestational weeks 5-23. (E) *GRB2* expression in cell types of prenatal human brain transcriptomics atlas. (F) *GRB2* expression in the adult human brain single-nucleus RNA sequencing data.

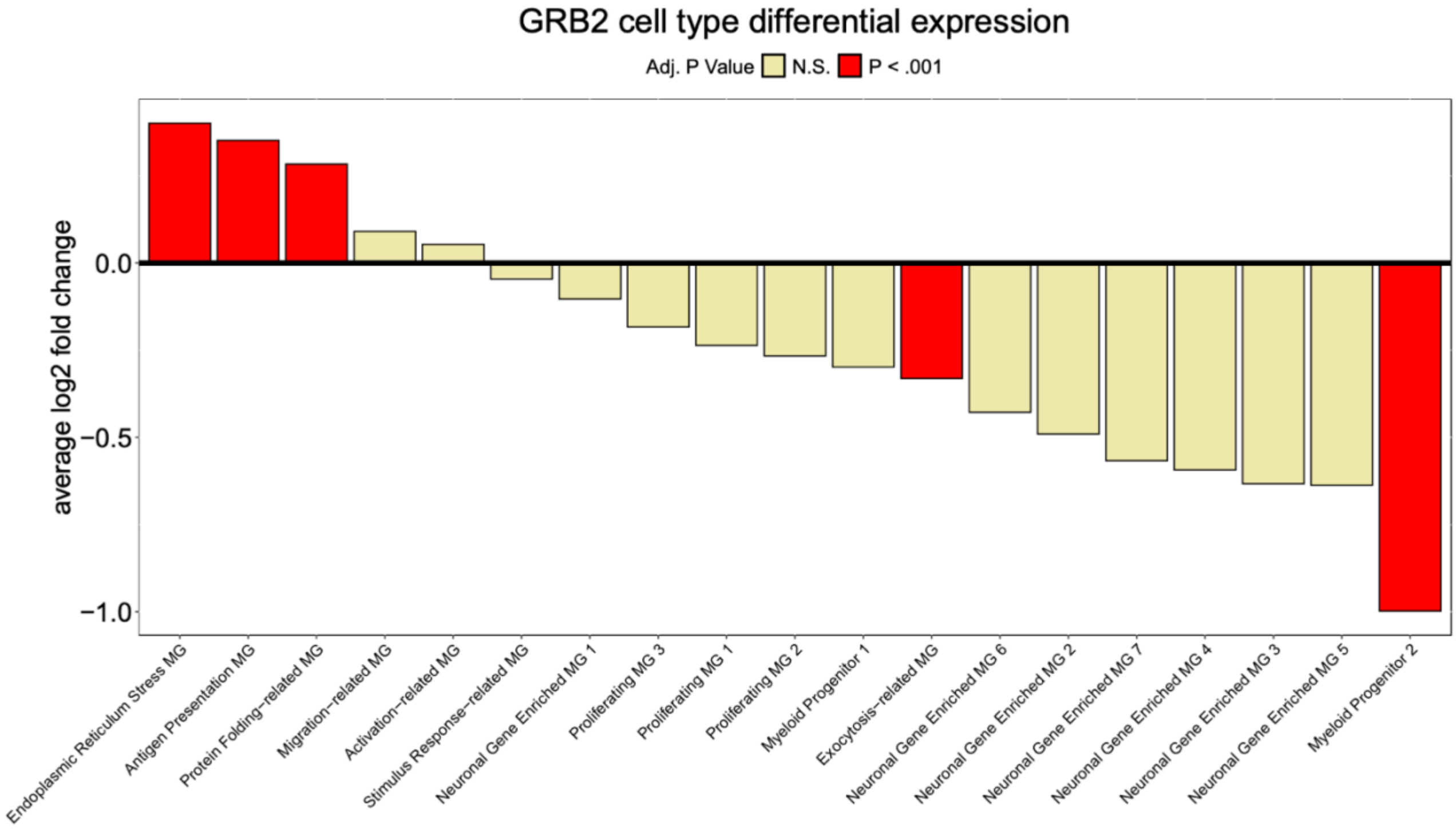

Supplementary Figure 3: *GRB2* differential expression across microglial subclusters. Clusters labeled by biological function based on top differentially expressed genes.

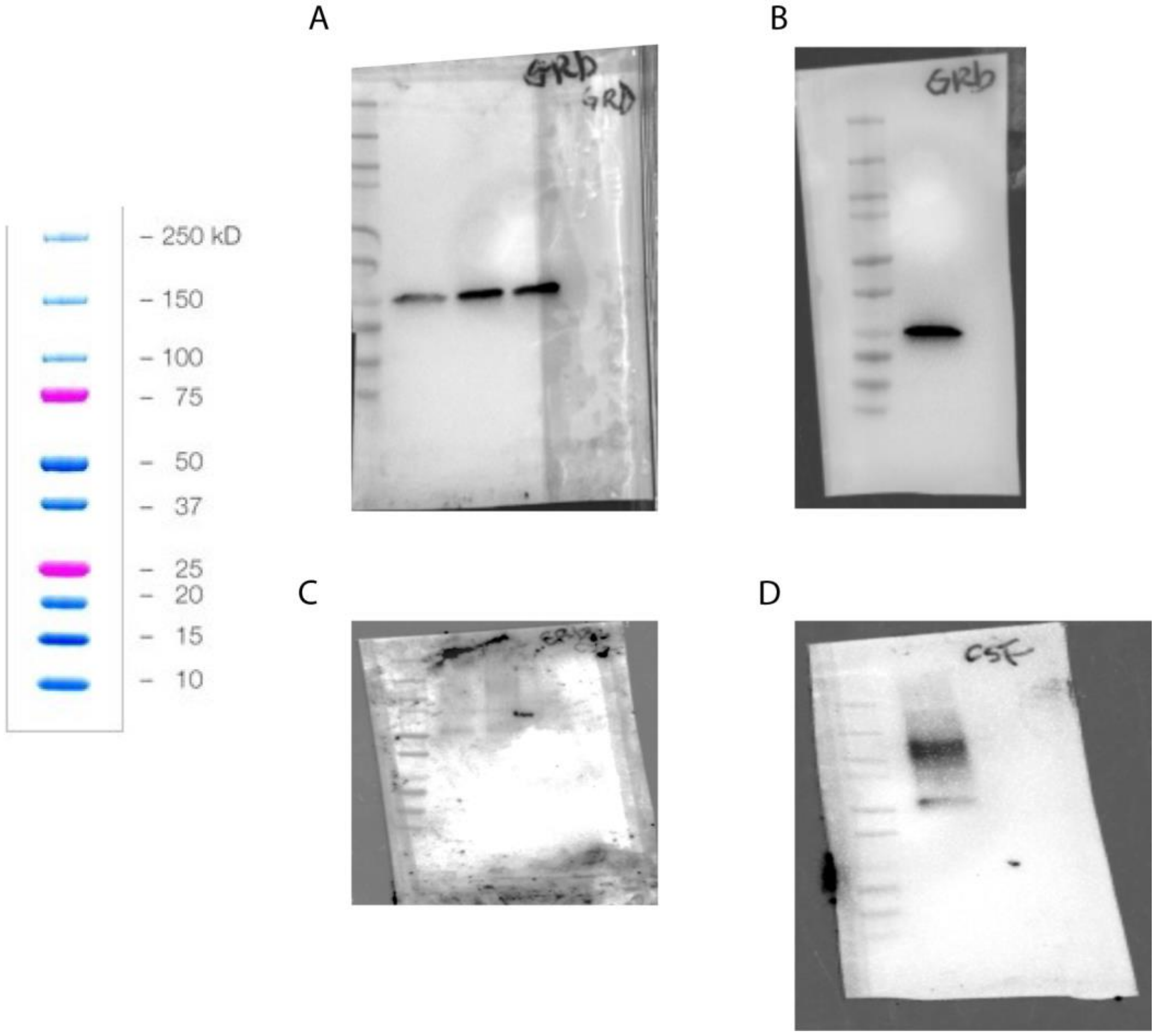

Supplementary Figure 4: Source data. A) Source blot corresponding to Figure 2G, top lane. B) Source blot corresponding to Figure 2G, third lane down from top. C) Source blot corresponding to Figure 2G, second lane down from top. D) Source blot corresponding to Figure 2G, fourth lane down from top.

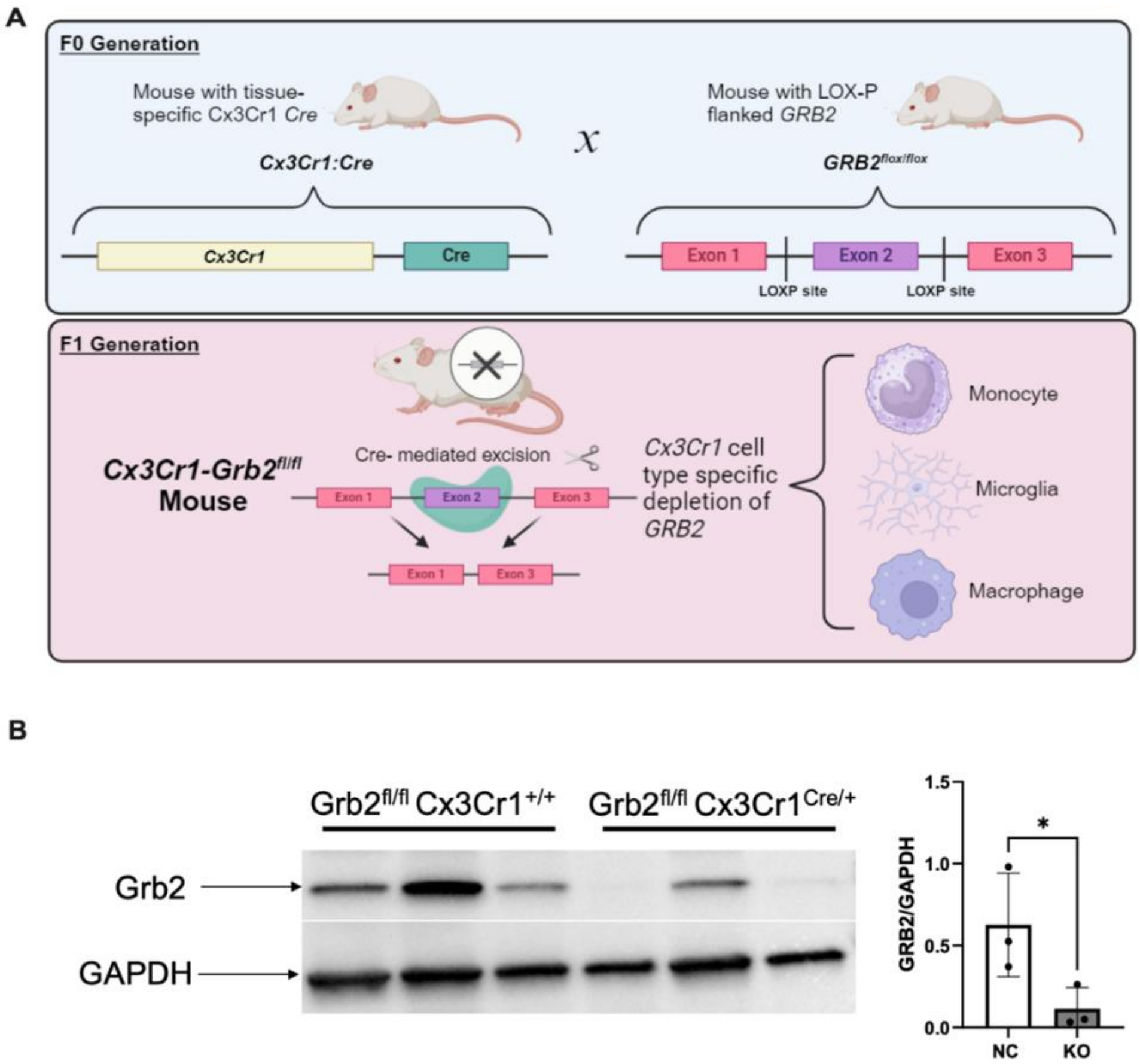

Supplementary Figure 5: Methodology and confirmation of microglia-specific knockout of GRB2 in mouse model. (A) Schematic of cross between *Cx_3Cr1*:Cre mice and conditional *$Grb2^{fl/fl}$* mice to generate progeny *Cx_3Cr1-$Grb2^{fl/fl}$* mice with functional *Grb2* deletion from all mononuclear phagocytes, including microglia. (B) Immunoblot for Cx3Cr1+ peritoneal macrophages of $Grb2^{fl/fl}$ $Cx3Cr1^{+/+}$ mice (negative control, NC) and $Grb2^{fl/fl}$ $Cx3Cr1^{cre/+}$ mice (KO), done with an anti-Grb2 antibody and an anti-GAPDH antibody as a control. Quantification of protein expression normalized to the level of GAPDH; means ± SEM, n = 3 samples per group, *$P < 0.05$.

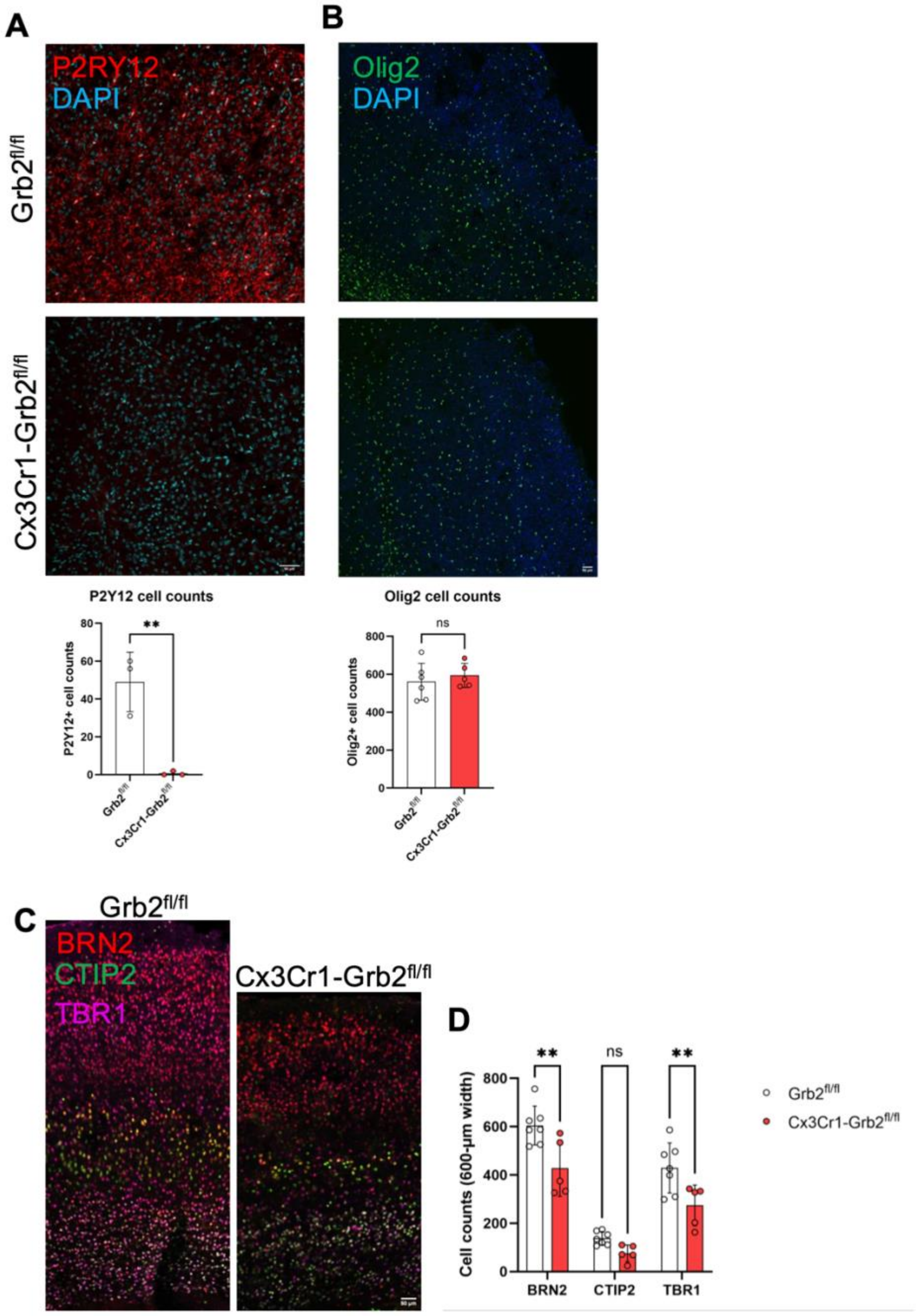

Supplementary Figure 6: Characterization of microglia-specific knockout of GRB2 in the mouse brain. (A–B) Representative immunostaining of P2Y12 (A, microglia marker) and Olig2 (B, oligodendrocyte marker) in brains of *Grb2*$^{fl/fl}$ and *Cx3cr1-Grb2*$^{fl/fl}$ mice. Quantification of P2RY12^{+} cells (A), Olig2^{+} cells (B), and GFAP fluorescence intensity (C) is shown below the respective images. n = 3 (A), n = 5–6 (B), n = 5 (C); unpaired Student's t-test. (D) Immunostaining of BRN2 (layer II–III cortical neurons), BCL11B/CTIP2 (layer V cortical neurons), and TBR1 (layer VI cortical neurons) in cortices of *Grb2*$^{fl/fl}$ and *Cx3cr1-Grb2*$^{fl/fl}$ mice. (E) Quantification of cell numbers from panel D. n = 5–7; two-way ANOVA followed by Bonferroni's multiple comparisons test. Scale bar: 50 µm.

Supplementary Table 1. Summary of Human and Mouse Evidence Linking CSF1R and PTPN11 (SHP2) Mutations to Hydrocephalus and/or Ventriculomegaly.

Gene	Species	Type of Study / Model	Phenotype Description (Hydrocephalus/Ventriculomegaly)	OMIM	Citation
CSF1R	Human	Case report (n=1)	Adult-onset leukoencephalopathy (HDLS) with striking ventricular enlargement misdiagnosed as NPH.<br>**Variant:** Heterozygous mutation c.2552T>C (p.L851P)	221820	Wang *et al.* 2019 (PMID:31093799)
		Case series (n=7)	Recessive CSF1R mutations (BANDDOS): congenital hydrocephalus with ventriculomegaly, callosal agenesis, and cerebellar hypoplasia.<br>**Variants:** c.395C>T (p.Pro132Leu), c.1441C>T (p.Gln481*), c.1859119G>A (p.Ser620delins40), c.1879_1881del (p.Lys627del), c.1969þ115_1969þ116del (p.Pro658Serfs*24)	618476<br>224300<br>265990	Guo *et al.* 2019 (PMID:30982609)
		Case series (n=2)	Ventriculomegaly with callosal agenesis and pontocerebellar hypoplasia due to near-complete absence of microglia.<br>**Variants:** c.1754-1G>C, c.1929C>A (p.His643Gln)	618476	Oosterhof *et al.* 2019 (PMID:30982608)
		Case series (n=2)	Dandy-Walker malformation, occipital ventriculomegaly (colpocephaly), callosal agenesis, periventricular calcifications.<br>**Variant:** c.1620T>A (p.Tyr540*)	220200	Monies *et al.* 2017 (PMID:28383543)
		Case report (n=1)	Childhood-onset neurodegeneration with cerebral atrophy, ex-vacuo ventricular dilatation, and periventricular white matter abnormalities; confirmed biallelic hypomorphic mutation (p.T833M).<br>**Variants:** Chr5:149435645G > A /c.2498C > T /p.(Thr833Met) /rs780804532	164770	Tamhankar *et al.* 2020 (PMID:32464672)
		Case series (n=7)	Ventricular enlargement (lateral ventricles), thinning of corpus callosum, white matter loss with frontal predominance, progressive cerebral atrophy.<br>**Variants:** c.2294G.A/p.G765D, c.2342C.A/p.A781E, c.2470C.T/p.P824S, c.244211G.T, c.2060_2061insT/p.S688EfsX13, c.2381T.C/p. I794T	164770	Konno *et al.* 2014 (doi:10.1212/WNL.00 00000000000046)
		Cohort study (n=26)	Dilation of lateral ventricles, thinning of the corpus callosum, white matter lesions, and calcifications in white matter.	164770	Konno *et al.* 2017 (doi:10. 1111/ene.13125)
		Observat ional study (n=1)	Misdiagnosed NPH; later found to have CSF1R mutation (HDLS).	164770	Rademakers *et al.* 2012 (doi:10.1038/ng.1027)
		Cohort Study (n=13)	Brain abnormalities included white matter changes, calcifications, agenesis of corpus callosum, ventriculomegaly, Dandy-Walker complex, and cortical abnormalities.	618476<br>220200<br>221820	Dulski *et al.* 2023 (PMID:37349768)
		Case report (n=1)	Brain MRI disclosed frontoparietal lobe atrophy and ventricular dilatation with high-intensity lesions in the cerebral deep white matter<br>**Variant:** de novo K793T mutation	221820	Kondo *et al.* 2013 (PMID:23411710)

		Case report (n=1)	Brain MRI showed cerebral atrophy, enlarged lateral ventricles, thinning of the corpus callosum, and leukoencephalopathy in the frontal-parietal lobes.<br>**Variant:** c.2330G>A/p.R777Q	221820	Yokote *et al.* 2020 (PMID:32435043)
		Familial case series (n=1)	Severe diffuse white matter atrophy and dilation of lateral and third ventricles. Hypoperfusion of cerebral cortices.<br>**Variant:** heterozygous c.653C>Y	221820	Riku *et al.* 2014 (PMID: 25383640)
		Case report (n=1)	Progressive confluent periventricular white matter lesions and enlarged lateral ventricles.<br>**Splicing mutation**: c.1858+1G>T (exon 13 skipping)	221820	Yang *et al.* 2019 (doi: 10.3389/fgene.2019.00491)
		Case series (n=3)	Global atrophy and enlarged ventricles.<br>**Variant:** c.2342C>T (p.A781V), exon 18	221820	Battisti *et al.* 2014 (PMID:24532199)
		Case report (n=1)	Multifocal leukoencephalopathy, thinning of corpus callosum, ventriculomegaly, parieto-occipital involvement.<br>**Variant:** c.2539G>A (p.Glu847Lys), exon 19	221820	Di Donato *et al.* 2015 (PMID:26401554)
		Case report (n=2)	Diffuse brain atrophy, ventricular enlargement, bilateral white matter changes.<br>**Variant:** c.2264T>C (p.L755P)	221820	Du *et al.* 2021 (PMID:30617447)
		Case report (n=1)	Severe cerebral atrophy and lateral/third ventricular enlargement.<br>**Variant:** c.2442+2T>C, exon 18	221820	Kawakami *et al.* 2016 (PMID:27423618)
		Case series (n=1)	Frontal atrophy, periventricular white matter lesions, and lateral ventricle dilation.<br>**Variant:** c.2381T>C (p.I794T)	221820	Kim *et al.* 2017 (PMID:29044417)
		Case report (n=1)	Confluent white matter lesions in frontoparietal lobes, ventriculomegaly.<br>**Variant:** c.2539G>A (p.E847K), exon 19	221820	Kim *et al.* 2019 (PMID:30853829)
		Case report (n=1)	Frontal-predominant diffuse leukoencephalopathy, thinning of anterior corpus callosum, ventriculomegaly.<br>**Variant:** c.2350G>A (p.V784M), exon 18	221820	La Piana *et al.* 2014 (PMID:25012610)
		Case report (n=1)	Severe corpus callosum thinning and ventricular dilation. Persistent diffusion-restricted lesions.<br>**Variant:** c.2319+1C>A, intron 16	221820	Leng *et. al.* 2019 (PMID:31632577)
		Case report (n=1)	Diffuse frontal white matter atrophy and progressive ventriculomegaly<br>**Variant:** c.2473G>A (p.E825K), exon 19	221820	Lynch *et al.* 2016 (PMID:25935893
		Case series (n=6)	Periventricular and subcortical WM lesions, ventriculomegaly, callosal thinning.<br>**Variants:** c.2546_2548delTCT, c.2381T>C (p.I794T), c.2563C>A	221820	Mao *et. al.* 2020 (PMID:31705326)
		Case report (n=1)	Frontal and occipital white matter hyperintensities, enlarged frontal horns, diffuse atrophy.<br>**Variant:** c.2345G>A (p.R782H), exon 18	221820	Nicholson *et al.* 2013 (PMID: 23408870)
		Case report (n=1)	Mild atrophy, periventricular calcifications, ventriculomegaly, and gliosis.<br>**Variant:** c.2467C>T (p.Ala823Val), exon 19	221820	Terasawa *et al.* 2013 (PMID:24094860)

		Case report (n=1)	Diffuse cerebral atrophy with frontal predominance and lateral/third ventricle enlargement.<br>**Variant:** c.2552T>C (p.L851P), exon 19	221820	Wang M., Zhang, X. 2019 (PMID:31093799)
		Familial case series (n=1)	Global atrophy, enlarged ventricles, posterior callosal thinning.<br>**Variant:** c.2342C>T (p.A781V), exon 18	221820	Ahmed *et al.* 2013 (PMID: 23816250)
		Case series (n=1)	Enlarged ventricles with frontal-predominant WM lesions.<br>**Variant:** c.1990G>A (novel heterozygous mutation), exon 15	221820	Eichler *et al.* 2016 (PMID: 27190017)
	**Mouse**	KI	*Csf1r*$^{E631K/\mathrm{E631K}}$ homozygotes: absent microglia, severe ventriculomegaly, callosal hypoplasia, hydrocephalus, perinatal lethality.	-	Stables *et al.* 2022 (PMID: 35333324)
		Constitutive KO	*Csf1r*$^{-/-}$ mice: lethal hydrocephalus with ventricular dilation and cortical thinning.	-	Erblich *et al.* 2011 (PMID:22046273)
		Heterozygous KO	*Csf1r*$^{+/-}$ adult mice: ventriculomegaly, callosal thinning, white matter degeneration.	-	Chitu *et al.* 2015 (PMID:25497733)
		KO	Enlarged ventricles, reduced cortex, global brain size reduction.	-	Nandi *et al.* 2012 (doi:10.1016/j.ydbio.2012.03.026)
PTPN11 **(SHP2)**	**Human**	Case report (n=1)	Noonan syndrome with obstructive tetraventricular hydrocephalus due to a fourth ventricle outlet obstruction (diverticulum of Luschka); resolved after endoscopic ETV.	163950	Spennato *et al.* 2019 (PMID: 30822582)
		Case series (prenatal; n=1)	Prenatal diagnosis of Noonan syndrome with external hydrocephalus (enlarged subarachnoid spaces) from 19 weeks gestation.<br>**Variant:** c.124A>G, p.Thr42Ala; chr12-112884189 A>G,<br>NM_002834.5, rs397507501	163950	Elekes *et al.* 2025 (doi:10.3390/jcm14113973)
		Case report (prenatal, n=1)	Fetal Noonan syndrome: external hydrocephalus on MRI.<br>Variant: *PTPN11*(p.Asn308Ser)	163950	Mastromoro *et al.*, 2021 (PMID:34075583)
	**Mouse**	KI	*Ptpn11*^{E76K} pan-neuronal knock-in: communicating hydrocephalus via ependymal/ciliary dysfunction; rescued by SHP2 C459S.	-	Zheng *et al.* 2018 (PMID: 29559584)

Patent list for Benjamin P. Kleinstiver

Type Code - IP	Status Code - IP	Country - IP	Application Number - IP	Application Date - IP	Patent Number - IP	Issue Date - IP
Provisional	Converted	United States of America	62/965,709	1/24/20		
PCT	Nationalized	International Bureau of the World Intellectual Property Organization (WIPO)	PCT/US2021/014900	1/25/21		
Utility	Allowed	United States of America	17/157,708	1/25/21		
PCT	Pending	European Patent Office (EPO)	21744681.4	1/25/21		
Continuation	Pending	United States of America	19/218,042	6/4/25		
Provisional	Converted	United States of America	62/965,645	1/24/20		
PCT	Nationalized	International Bureau of the World Intellectual Property Organization (WIPO)	PCT/US2021/014887	1/25/21		
PCT	Allowed	United States of America	17/794,520	7/21/22		
PCT	Pending	European Patent Office (EPO)	21744778.8	1/25/21		
Utility	Issued	United States of America	17/157,805	1/25/21	12,264,341	4/1/25
Provisional	Converted	United States of America	62/965,671	1/24/20		
PCT	Nationalized	International Bureau of the World Intellectual Property Organization (WIPO)	PCT/US2021/014933	1/25/21		
PCT	Pending	European Patent Office (EPO)	21744577.4	1/25/21		
Continuation	Pending	United States of America	19/064,527	1/25/21		
Provisional	Converted	United States of America	63/135,471	1/8/21		
PCT	Pending	International Bureau of the World Intellectual Property Organization (WIPO)	PCT/US2022/011823	1/10/22		
PCT	Pending	United States of America	18/271,190	7/6/23		
PCT	Pending	European Patent Office (EPO)	22737254.7	1/10/22		
Provisional	Converted	United States of America	63/411,367	9/29/22		
Provisional	Converted	United States of America	63/459,463	4/14/23		
PCT	Nationalized	International Bureau of the World Intellectual Property Organization (WIPO)	PCT/US2023/075589	9/29/23		

PCT	Pending	United States of America	19/116,185	3/27/25		
PCT	Pending	European Patent Office (EPO)	23874010.4	9/29/23		
Provisional	Converted	United States of America	63/285,857	12/3/21		
Provisional	Converted	United States of America	63/291,264	12/17/21		
Provisional	Converted	United States of America	63/411,735	9/30/22		
PCT	Nationalized	International Bureau of the World Intellectual Property Organization (WIPO)	PCT/US2022/051639	12/2/22		
PCT	Pending	United States of America	18/715,209	5/31/24		
PCT	Pending	Canada	3,241,474	12/2/22		
PCT	Pending	European Patent Office (EPO)	22902222.3	12/2/22		
PCT	Pending	Japan	2024-532847	12/2/22		
Provisional	Converted	United States of America	63/400,443	8/24/22		
PCT	Nationalized	International Bureau of the World Intellectual Property Organization (WIPO)	PCT/US2023/072851	8/24/23		
PCT	Pending	United States of America	19/106,116	2/24/25		
PCT	Pending	European Patent Office (EPO)	23858315.7	8/24/23		
Provisional	Converted	United States of America	63/495,047	4/7/23		
Provisional	Converted	United States of America	63/581,592	11/10/23		
PCT	Nationalized	International Bureau of the World Intellectual Property Organization (WIPO)	PCT/US2024/023562	4/8/24		
PCT	Pending	United States of America	19/473,420	10/7/25		
PCT	Pending	European Patent Office (EPO)	24785929.1	4/8/24		
PCT	Pending	China	202480037734.9	4/8/24		
Provisional	Converted	United States of America	63/458,519	4/11/23		
Provisional	Converted	United States of America	63/590,283	10/13/23		
PCT	Nationalized	International Bureau of the World Intellectual Property Organization (WIPO)	PCT/US2024/024078	4/11/24		
PCT	Pending	United States of America	19/474,100	10/9/25		
Provisional	Converted	United States of America	63/497,943	4/24/23		

PCT	Nationalized	International Bureau of the World Intellectual Property Organization (WIPO)	PCT/US2024/025880	4/23/24		
PCT	Pending	United States of America	19/478,664	10/24/25		
Provisional	Converted	United States of America	63/375,008	9/8/22		
PCT	Nationalized	International Bureau of the World Intellectual Property Organization (WIPO)	PCT/GB2023/052315	9/7/23		
PCT	Pending	European Patent Office (EPO)	23786121.6	9/7/23		
PCT	Pending	United States of America	19/109,672	9/7/23		
PCT	Pending	Japan	2025-514212	9/7/23		
PCT	Pending	Canada	3,266,991	9/7/23		
PCT	Pending	China	202380077365.1	9/7/23		
Provisional	Converted	United States of America	63/589,594	10/11/23		
Provisional	Converted	United States of America	63/638,312	4/24/24		
PCT	Nationalized	International Bureau of the World Intellectual Property Organization (WIPO)	PCT/US2024/051099	10/11/24		
PCT	Pending	United States of America	19/527,809	4/10/26		
PCT	Pending	Canada	3,307,843	10/11/24		
PCT	Pending	Australia	2024357356	10/11/24		
PCT	Pending	Korea, Republic of	10-2026-7014167	10/11/24		
PCT	Pending	Japan	2026-521835	10/11/24		
PCT	Pending	European Patent Office (EPO)	24878172.6	10/11/24		
PCT	Pending	China	202480078465.0	10/11/24		
Provisional	Converted	United States of America	63/546,914	11/1/23		
PCT	Nationalized	International Bureau of the World Intellectual Property Organization (WIPO)	PCT/US2024/054176	11/1/24		
PCT	Pending	United States of America	19/583,849	4/30/26		
Provisional	Converted	United States of America	63/624,377	1/24/24		
PCT	Nationalized	International Bureau of the World Intellectual Property Organization (WIPO)	PCT/IB2025/050835	1/24/25		
PCT	Pending	United States of America	19/609,352	7/24/26		

PCT	Pending	Japan	TBA	1/24/25		
PCT	Pending	European Patent Office (EPO)	25744822.5	1/24/25		
Provisional	Converted	United States of America	63/644,379	5/8/24		
PCT	Pending	International Bureau of the World Intellectual Property Organization (WIPO)	PCT/US2025/028520	5/8/25		
Provisional	Converted	United States of America	TBA	4/1/25		
Provisional	Converted	United States of America	63/555,774	2/20/24		
PCT	Nationalized	International Bureau of the World Intellectual Property Organization (WIPO)	PCT/CA2025/050213	2/20/25		
PCT	Pending	United States of America	19/781,813	2/20/25		
Provisional	Converted	United States of America	63/639,553	4/26/24		
PCT	Pending	International Bureau of the World Intellectual Property Organization (WIPO)	PCT/US2025/026694	4/29/25		
Provisional	Pending	United States of America	63/935,618	12/10/25		
Provisional	Converted	United States of America	63/707,548	10/15/24		
PCT	Pending	International Bureau of the World Intellectual Property Organization (WIPO)	PCT/US2025/051052	10/15/25		
Provisional	Converted	United States of America	63/674,450	7/23/24		
PCT	Pending	International Bureau of the World Intellectual Property Organization (WIPO)	PCT/US2025/038938	7/23/25		
Provisional	Converted	United States of America	63/795,815	4/28/25		
PCT	Pending	International Bureau of the World Intellectual Property Organization (WIPO)	PCT/US2026/025602	4/28/26		
Provisional	Pending	United States of America	64/063,674	5/12/26		
Provisional	Pending	United States of America	64/058,633	5/6/26		
Provisional	Converted	United States of America	62/058,456	10/1/14		

PCT	Nationalized	International Bureau of the World Intellectual Property Organization (WIPO)	PCT/US2015/053417	10/1/15		
Divisional	Issued	European Patent Office (EPO)	20216630.2	3/3/16	3858990	5/6/26
Divisional	Issued	France	20216630.2	3/3/16	3858990	5/6/26
Divisional	Issued	Germany	20216630.2	3/3/16	3858990	5/6/26
Divisional	Issued	United Kingdom	20216630.2	3/3/16	3858990	5/6/26
Continuation	Issued	United States of America	17/010,107	9/2/20	12,180,520	12/31/24
Divisional	Issued	Japan	2023-044993	3/3/16	7585373	11/8/24
Divisional	Issued	Australia	2022201915	3/3/16	2022201915	10/10/24
Continuation	Issued	United States of America	17/456,092	11/22/21	11,859,220	1/2/24
PCT	Issued	Korea, Republic of	10-2017-7026620	3/3/16	10-2598856	11/1/23
Continuation	Issued	United States of America	17/012,697	9/4/20	11,220,678	1/11/22
PCT	Issued	United Kingdom	16759521.4	3/3/16	3265559	1/6/21
PCT	Issued	Germany	16759521.4	3/3/16	3265559	1/6/21
PCT	Issued	France	16759521.4	3/3/16	3265559	1/6/21
PCT	Issued	European Patent Office (EPO)	16759521.4	3/3/16	3265559	1/6/21
Divisional	Issued	United States of America	16/687,396	11/18/19	10,808,233	10/20/20
Continuation	Issued	United States of America	15/664,873	7/31/17	10,767,168	9/8/20
Continuation	Issued	United States of America	15/935,334	3/26/18	10479982	11/19/19
Utility	Issued	United States of America	15/060,448	3/3/16	10,202,589	2/12/19
Utility	Issued	United States of America	15/060,424	3/3/16	9,944,912	4/17/18
Continuation	Issued	United States of America	15/208,228	7/12/16	9,926,545	3/27/18
Continuation	Issued	United States of America	15/208,461	7/12/16	9,752,132	10/27/16
Divisional	Allowed	Japan	2020-214920	3/3/16		
Provisional	Converted	United States of America	62/127,634	3/3/15		
Provisional	Converted	United States of America	62/165,517	5/22/15		
Provisional	Converted	United States of America	62/239,737	10/9/15		
Provisional	Converted	United States of America	62/258,402	11/20/15		
PCT	Nationalized	International Bureau of the World Intellectual Property Organization (WIPO)	PCT/US2016/020756	3/3/16		
PCT	Pending	Australia	2016226077	3/3/16		
PCT	Allowed	Canada	2,978,314	3/3/16		
PCT	Allowed	China	201680024041.1	3/3/16		
PCT	Issued	Japan	2017-546196	3/3/16	6817215	
PCT	Pending	Hong Kong	18108541.7	3/3/16		

Continuation	Pending	United States of America	18/507,709	11/13/23		
Divisional	Allowed	Korea, Republic of	10-2023-7037790	3/3/16		
Divisional	Pending	Japan	2024-103113	3/3/16		
Divisional	Pending	European Patent Office (EPO)	26169982.1	3/3/16		
PCT	Issued	Canada	2,996,888	8/26/16	2,996,888	1/27/26
Divisional	Issued	Japan	2023-125901	5/26/16	7731943	8/22/25
Divisional	Issued	China	202210550934.7	8/26/16	CN 114875012 B	2/18/25
Divisional	Issued	Australia	2022203146	8/26/16	2022203146	12/12/24
PCT	Issued	Korea, Republic of	10-2018-7008311	8/26/16	10-2668608	5/24/24
PCT	Issued	India	201817009799	8/26/16	514743	2/23/24
Divisional	Issued	Japan	2020-193171	8/26/16	7074827	5/16/22
PCT	Issued	United Kingdom	16842722.7	8/26/16	3341477	3/23/22
PCT	Issued	Germany	16842722.7	8/26/16	3341477	3/23/22
PCT	Issued	France	16842722.7	8/26/16	3341477	3/23/22
PCT	Issued	European Patent Office (EPO)	16842722.7	8/26/16	3341477	3/23/22
Utility	Issued	United States of America	16/734,935	1/6/20	11,060,078	7/13/21
PCT	Issued	Japan	2018-510914	8/26/16	6799586	11/25/20
Continuation	Issued	United States of America	16/155,808	10/9/18	10,633,642	4/28/20
Continuation	Issued	United States of America	15/935,299	3/26/18	10526591	1/7/20
Continuation	Issued	United States of America	15/369,533	10/5/16	10,093,910	10/9/18
Cont-in-Part	Issued	United States of America	15/249,756	8/29/16	9,926,546	3/27/18
Utility	Issued	United States of America	15/015,947	2/4/16	9,512,446	12/6/16
Divisional	Allowed	Japan	2019-153881	8/26/16		
PCT	Pending	Australia	2016316845	8/26/16		
Divisional	Allowed	Japan	2020-131422	8/26/16		
Continuation	Pending	United States of America	17/331,883	5/27/21		
Divisional	Allowed	Japan	2021-145911	8/26/16		
Divisional	Pending	European Patent Office (EPO)	22156340.6	8/26/16		
Divisional	Pending	India	202218046918	8/26/16		
Provisional	Converted	United States of America	62/211,553	8/28/15		
Provisional	Converted	United States of America	62/216,033	9/9/15		
Provisional	Converted	United States of America	62/258,280	11/20/15		
Provisional	Converted	United States of America	62/271,938	12/28/15		
PCT	Nationalized	International Bureau of the World Intellectual Property Organization (WIPO)	PCT/US2016/049147	8/26/16		
PCT	Allowed	China	201680063266.8	8/26/16		
Divisional	Pending	India	202418004424	8/26/16		

Divisional	Pending	Korea, Republic of	10-2024-7016635	8/26/16		
Divisional	Pending	India	202418041505	8/26/16		
Divisional	Pending	Canada	3,286,103	8/26/16		
Continuation	Issued	United States of America	17/450,588	10/12/21	12,649,913	6/9/26
Divisional	Issued	United States of America	17/450,593	10/12/21	12,590,299	3/31/26
Divisional	Issued	Australia	2023208113	7/25/17	2023208113	1/8/26
Utility	Issued	United States of America	15/659,499	7/25/17	11,168,313	11/9/21
Provisional	Converted	United States of America	62/366,976	7/26/16		
PCT	Nationalized	International Bureau of the World Intellectual Property Organization (WIPO)	PCT/US2017/043753	7/25/17		
PCT	Pending	Australia	2017302551	7/25/17		
PCT	Pending	Canada	3,031,414	7/25/17		
PCT	Pending	China	201780059001.5	7/25/17		
PCT	Pending	European Patent Office (EPO)	17835126.8	7/25/17		
Divisional	Pending	Australia	2025275207	7/25/17		
Divisional	Issued	Chile	202201524	4/23/18	73,555	6/23/26
Divisional	Issued	Chile	202102740	4/23/18	72157	11/19/25
Divisional	Issued	Chile	202102742	4/23/18	72158	11/19/25
PCT	Issued	Chile	201903004	4/23/18	71.134	6/3/25
Continuation	Issued	United States of America	17/244,738	4/29/21	12,173,339	12/24/24
PCT	Issued	European Patent Office (EPO)	18788179.2	4/23/18	3612551	9/4/24
PCT	Issued	Belgium	18788179.2	4/23/18	3612551	9/4/24
PCT	Issued	France	18788179.2	4/23/18	3612551	9/4/24
PCT	Issued	Germany	18788179.2	4/23/18	3612551	9/4/24
PCT	Issued	Italy	18788179.2	4/23/18	3612551	9/4/24
PCT	Issued	Netherlands	18788179.2	4/23/18	3612551	9/4/24
PCT	Issued	Spain	18788179.2	4/23/18	3612551	9/4/24
PCT	Issued	Switzerland	18788179.2	4/23/18	3612551	9/4/24
PCT	Issued	United Kingdom	18788179.2	4/23/18	3612551	9/4/24
Divisional	Issued	Australia	2022246445	4/23/18	2022246445	8/15/24
Utility	Issued	United States of America	15/960,271	4/23/18	11,591,589	2/28/23
PCT	Issued	Australia	2018254619	4/23/18	2018254619	11/3/22
PCT	Pending	Brazil	112019021719.3	4/23/18		
Divisional	Pending	Chile	202102739	4/23/18		
Divisional	Allowed	Chile	202102741	4/23/18		
Divisional	Pending	Chile	202201525	4/23/18		
Provisional	Converted	United States of America	62/488,426	4/21/17		
Provisional	Converted	United States of America	62/616,066	1/11/18		

PCT	Nationalized	International Bureau of the World Intellectual Property Organization (WIPO)	PCT/US2018/028919	4/23/18		
PCT	Pending	Canada	3059956	4/23/18		
PCT	Pending	China	201880042346.4	4/23/18		
Divisional	Pending	European Patent Office (EPO)	24197391.6	4/23/18		
Divisional	Pending	Brazil	1220240218348	4/23/18		
Divisional	Issued	United States of America	18/304,187	4/20/23	12,202,861	1/21/25
PCT	Issued	United States of America	16/606,680	10/18/19	11,667,677	6/6/23
PCT	Pending	European Patent Office (EPO)	18787309.6	4/23/18		
Provisional	Converted	United States of America	62/488,585	4/21/17		
PCT	Nationalized	International Bureau of the World Intellectual Property Organization (WIPO)	PCT/US2018/028898	4/23/18		
Divisional	Issued	Australia	2023278074	8/22/18	2023278074	1/8/26
Divisional	Issued	Australia	2025275152	8/22/18	2025275152	1/8/26
Continuation	Issued	United States of America	18/174,294	2/24/23	12,241,096	3/4/25
PCT	Issued	Japan	2020-511253	8/22/18	7581046	11/1/24
PCT	Issued	China	201880068905.9	8/22/18	CN111278450B	4/9/24
PCT	Issued	Australia	2018320865	8/22/18	2018320865	1/4/24
Continuation	Allowed	United States of America	17/674,558	2/17/22	11,624,058	4/11/23
Utility	Issued	United States of America	16/109,657	8/22/18	11,286,468	3/29/22
PCT	Pending	Canada	3,073,448	8/22/18		
PCT	Pending	European Patent Office (EPO)	18848977.7	8/22/18		
PCT	Pending	Brazil	1120200035963	8/22/18		
Provisional	Converted	United States of America	62/549,303	8/23/17		
Provisional	Converted	United States of America	62/641,687	3/12/18		
PCT	Nationalized	International Bureau of the World Intellectual Property Organization (WIPO)	PCT/US2018/047577	8/22/18		
Divisional	Pending	China	202410333226.7	8/22/18		
Divisional	Pending	Japan	2024-080681	8/22/18		
Divisional	Pending	Australia	2026204470	8/22/18		
Provisional	Converted	United States of America	62/515,938	6/6/17		
PCT	Nationalized	International Bureau of the World Intellectual Property Organization (WIPO)	PCT/US2018/036293	6/6/18		
