# Supplementary material for: Microglial GRB2 is essential for brain ventriculogenesis and cerebrospinal fluid homeostasis
Source: J Clin Invest. 2026 Sep 10;136(20):e185791. doi: 10.1172/JCI185791 (PMC13668624; doi:10.1172/JCI185791)
Supplement: Supplementary file 2 [file jci-136-185791-s076.pdf]

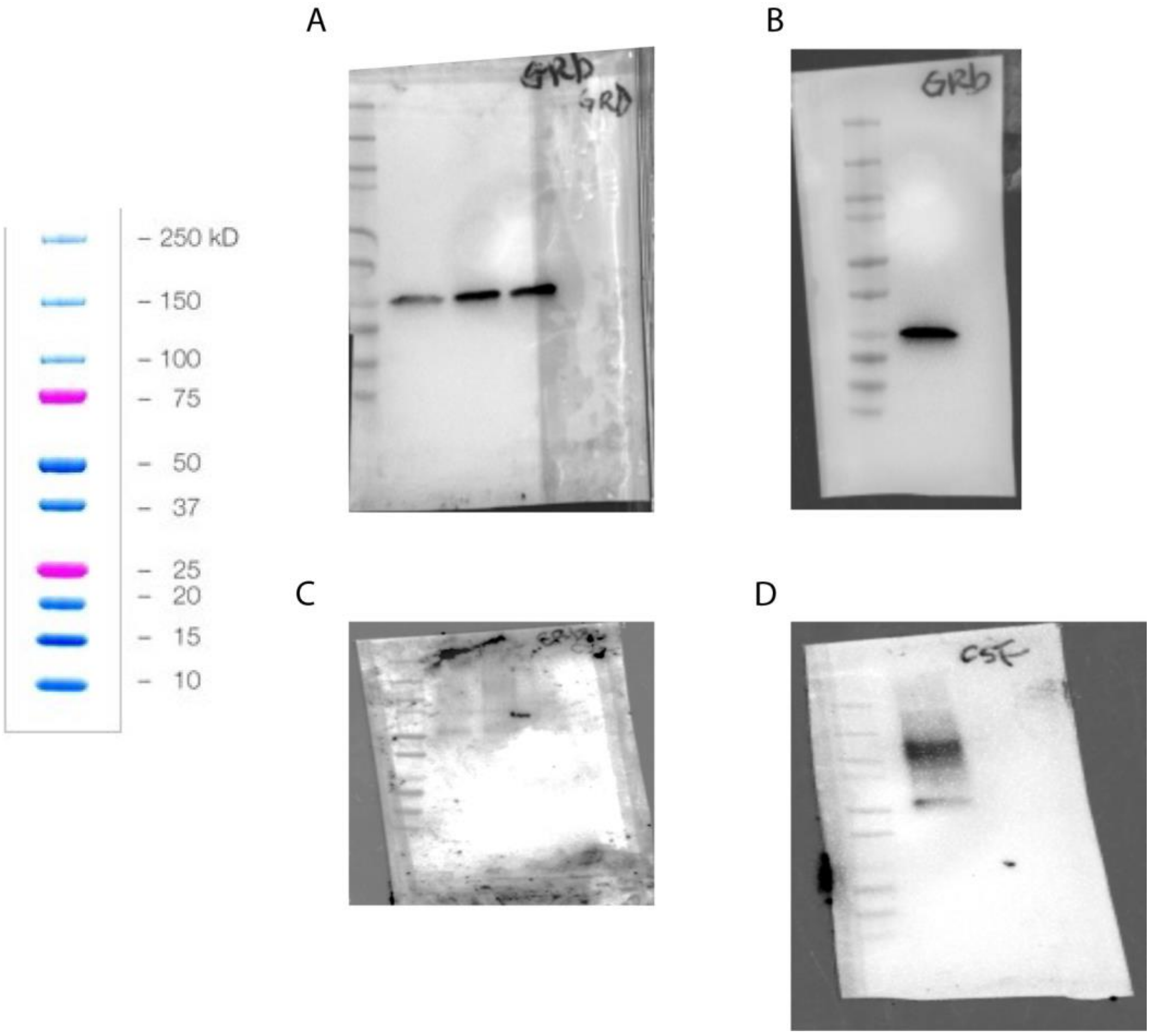

Supplementary Figure 4: Source data. A) Source blot corresponding to Figure 2G, top lane. B) Source blot corresponding to Figure 2G, third lane down from top. C) Source blot corresponding to Figure 2G, second lane down from top. D) Source blot corresponding to Figure 2G, fourth lane down from top.
